# Organocatalytic Asymmetric Synthesis of SynVesT-1, a Synaptic Density Positron Emission Tomography Imaging Agent

**DOI:** 10.1021/acs.joc.2c01895

**Published:** 2022-10-12

**Authors:** Holly McErlain, Euan B. McLean, Timaeus E. F. Morgan, Valeria K. Burianova, Adriana A. S. Tavares, Andrew Sutherland

**Affiliations:** † School of Chemistry, The Joseph Black Building, University of Glasgow, GlasgowG12 8QQ, U.K.; ‡ BHF-University Centre for Cardiovascular Science, University of Edinburgh, EdinburghEH16 4TJ, U.K.

## Abstract

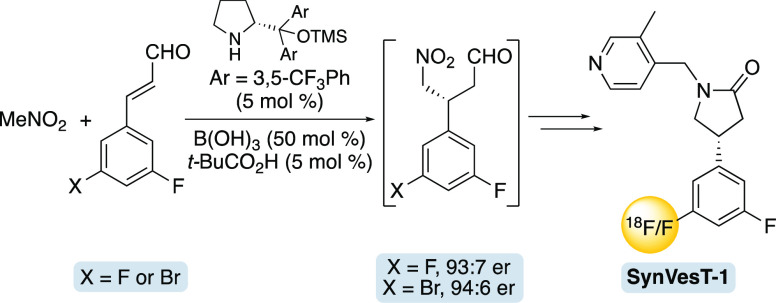

Heterocyclic nonacetamide ligands are used as positron emission tomography (PET) imaging agents of the synaptic vesicle glycoprotein 2A (SV2A), with potential applications in the diagnosis of various neuropsychiatric diseases. To date, the main synthetic strategy to access these optically active compounds has involved the racemic synthesis of a late-stage intermediate followed by the separation of the enantiomers. Here, we describe the use of iminium organocatalysis for the asymmetric synthesis of SynVesT-1, an important PET imaging agent of SV2A. The key step involved the conjugate addition of nitromethane with a cinnamaldehyde in the presence of the Jørgensen–Hayashi catalyst using the Merck dual acid cocatalyst system. Pinnick-type oxidation and esterification of the adduct was then followed by chemoselective nitro group reduction and cyclization using nickel borate. *N*-Alkylation of the resulting lactam then completed the seven-step synthesis of SynVesT-1. This approach was amenable for the synthesis of an organotin analogue, which following copper(II)-mediated fluoro-destannylation allowed rapid access to [^18^F]SynVesT-1.

## Introduction

**Figure 1:**
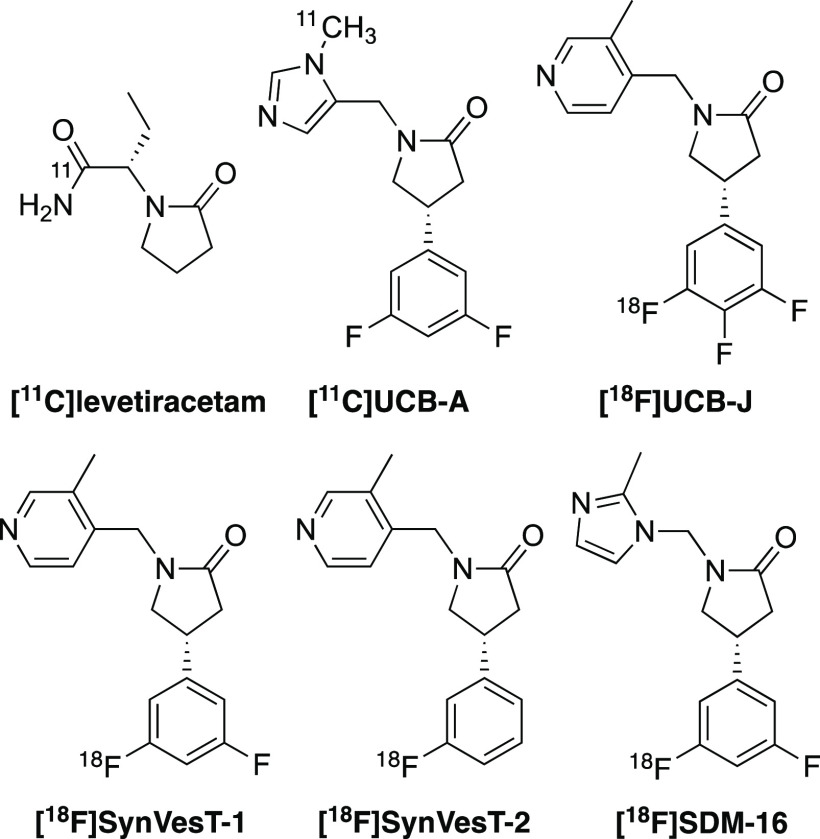
Structures of PET imaging agents of SV2A.

The synaptic vesicle glycoprotein 2A (SV2A) is a presynaptic transmembrane protein expressed in neurons across the brain and is critical for neural system functioning.^1^ It has been used as a biomarker of synaptic density and is associated with a variety of neurodegenerative and psychiatric disorders including Alzheimer’s disease, Parkinson’s disease, and schizophrenia. It is well established that SV2A is the binding target of the antiepileptic drug, levetiracetam (Keppra).^2^ To further understand the role of SV2A in neuropsychiatric diseases, a variety of radioligands have been developed and used in combination with positron emission tomography (PET) for preclinical and clinical imaging studies.^3^ Initial studies focused on the use of a radiolabeled version of [^11^C]levetiracetam (Figure 1),^4^ but the modest binding affinity for SV2A (*K*_i_ = 2.5 μM) prohibited the use of this tracer for in vivo imaging applications. This led to the discovery of heterocyclic nonacetamide ligands with low nanomolar affinity for SV2A.^5^ This class of compounds, which include UCB-A, SynVesT-1, and SDM-16, have subsequently been radiolabeled and used as PET tracers for preclinical and clinical imaging studies.^6−10^

**Scheme 1:**
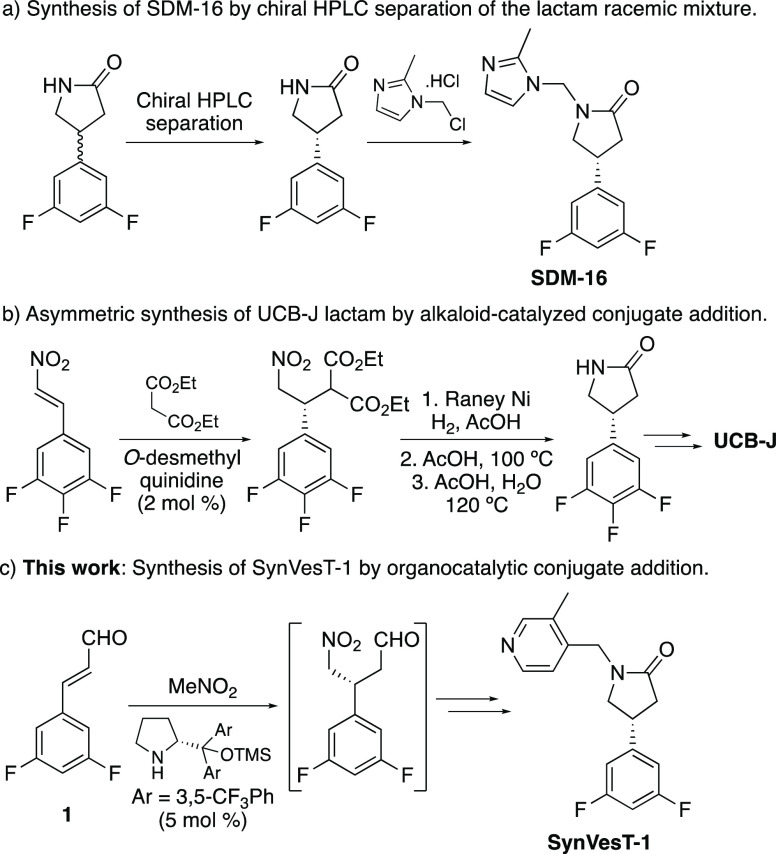
Synthetic Methods for the Preparation of SV2A Imaging Agents

During the development of these PET tracers, many of the in vivo studies reported that while the (*R*)-enantiomer of these compounds was highly effective for SV2A imaging, the (*S*)-enantiomers typically displayed low binding affinity, resulting in nonspecific, homogeneous brain uptake.^7b,8,10^ To access the more active (*R*)-enantiomer, the majority of studies employed a racemic synthesis of an advanced intermediate, typically a 4-aryl lactam, before using chiral high-performance liquid chromatography (HPLC) to isolate the desired (*R*)-stereoisomer (Scheme 1a).^6,7b,8−10^ The only example of an asymmetric synthesis was reported for the preparation of UCB-J.^7a^ An asymmetric conjugate addition of diethyl malonate with a β-nitrostyrene was performed in the presence of *O*-desmethyl quinidine, resulting in the (*R*)-enantiomer with 98% ee (Scheme 1b). Following the reduction of the nitro group, the synthesis of the 4-aryl lactam was then completed by heating in acetic acid over an extended period, which allowed intramolecular cyclization and then, hydrolysis and decarboxylation of the carboxylic ester.

We required access to [^18^F]SynVesT-1 and the nonradioactive analogue for the PET imaging of SV2A. Due to the limitations of previous syntheses of these PET imaging agents, particularly the requirement of chiral HPLC separation of an advanced intermediate and the loss of 50% of material, we sought to develop a short, asymmetric synthesis of SynVesT-1. We now report the synthesis of SynVesT-1 using asymmetric iminium organocatalysis for the key step (Scheme 1c). We also describe the use of this approach for the synthesis of an organotin precursor and the subsequent preparation of [^18^F]SynVesT-1.

## Results and Discussion

**Scheme 2:**
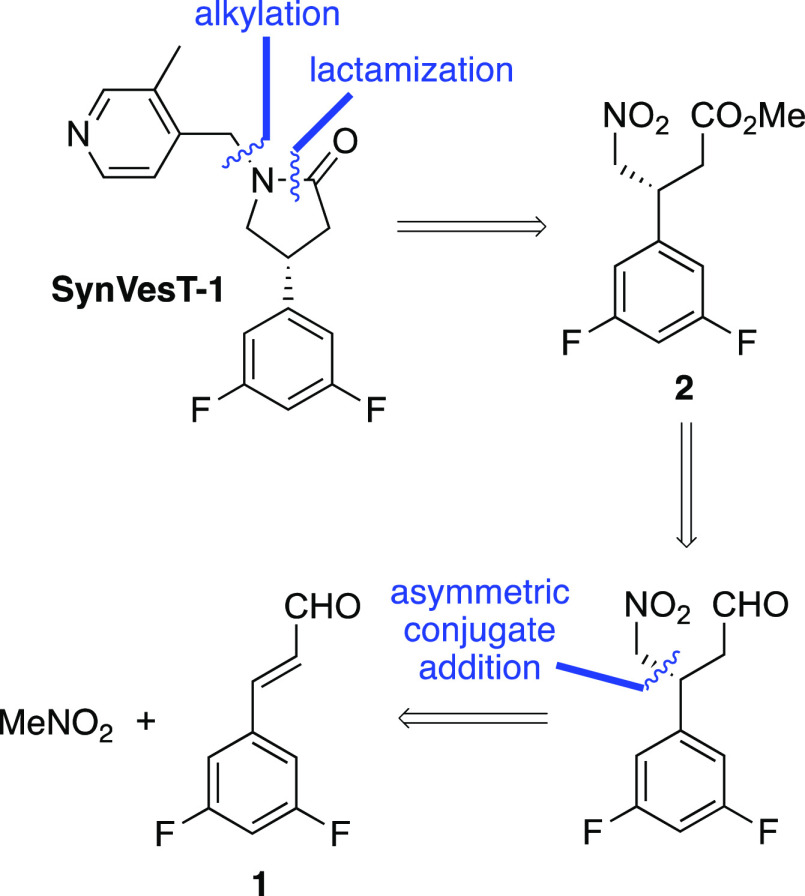
Retrosynthetic Analysis of SynVesT-1

As depicted in Scheme 2, we identified 3-aryl-4-nitrobutanoate **2** as the key intermediate, which following nitro group reduction and in situ lactamization, could undergo alkylation to access SynVesT-1. To avoid ester hydrolysis and decarboxylation under harsh acidic conditions employed during the synthesis of UCB-J,^7a^ we proposed an alternative approach to an enantioenriched 4-nitrobutanoate, involving the conjugate addition of nitromethane with cinnamaldehyde **1** using asymmetric iminium organocatalysis.^11^ Subsequent oxidation and esterification would then yield 3-aryl-4-nitrobutanoate **2**.

**Scheme 3:**
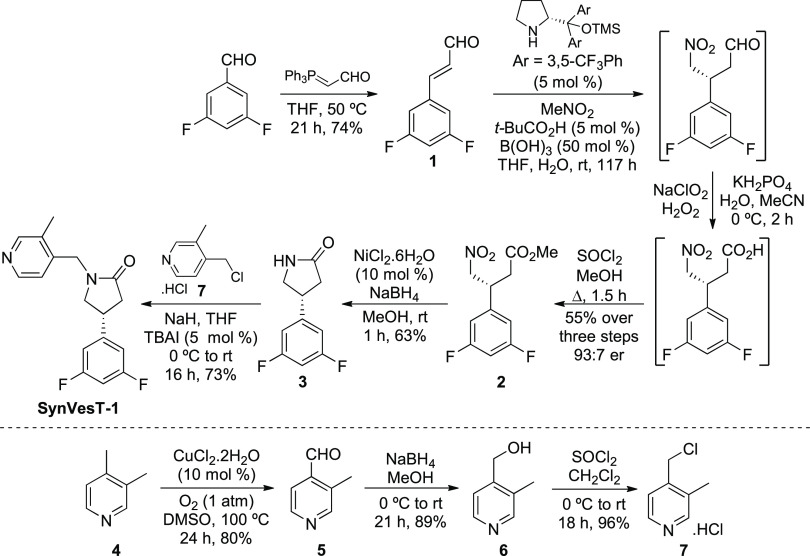
Asymmetric Synthesis of SynVesT-1 Isolated yields.

The asymmetric synthesis of SynVesT-1 began with the preparation of cinnamaldehyde **1** (Scheme 3). The reaction of commercially available 3,5-difluorobenzaldehyde with (triphenylphosphoranylidene)acetaldehyde gave the *E*-isomer of **1** as the sole product in 74% yield. Conditions for the key step and the conjugate addition of nitromethane with cinnamaldehyde **1** using asymmetric iminium organocatalysis were next developed. The use of Jørgensen–Hayashi catalysts^12^ has been widely screened for the conjugate addition of nitromethane with halogen containing cinnamaldehydes and found to produce the corresponding 3-aryl-4-nitrobutraldehydes with high yields and excellent enantioselectivity.^13^ For this reason, our initial studies used one of the Jørgensen–Hayashi catalysts in combination with conditions previously reported by Wang and co-workers, who developed an asymmetric conjugate addition of nitromethane with 4-chlorocinnamaldehyde for the synthesis of baclofen.^13a^ Their method used 20 mol % of a Jørgensen–Hayashi catalyst and the acidic cocatalyst PhCO_2_H. Using this general procedure (0 °C in ethanol), no reaction was observed with cinnamaldehyde **1**. However, at room temperature, the reaction did proceed and was complete after 46 h. Without purification, the desired unstable adduct was subjected to a Pinnick-type oxidation^14^ and subsequent esterification using thionyl chloride and methanol. This gave 3-aryl-4-nitrobutanoate **2** in 20% yield over the three steps and with an enantiomeric ratio of 93:7, as determined by chiral HPLC.^15^ During the large-scale synthesis of telcagepant, a calcitonin gene-related peptide receptor antagonist for the treatment of migraine, researchers at Merck found that the use of these conditions with a 2,3-difluorocinnamaldehyde analogue also produced several acetal byproducts, formed by the reaction of the starting material and product with the alcohol solvent.^16^ To circumvent this issue, they found that the use of aqueous tetrahydrofuran (THF) and the “cocktail” catalyst system of boric acid and pivalic acid helped suppress the formation of side-products. Using this procedure with cinnamaldehyde **1**, it was found that while the reaction took nearly 5 days to reach completion, nuclear magnetic resonance (NMR) spectroscopy showed clean and high conversion (95%) to the product. Oxidation and esterification as described before, gave **2** in an improved 55% yield over the three steps and, despite using a lower loading of the Jørgensen–Hayashi catalyst (5 mol %) during the conjugate addition reaction, the enantiomeric ratio of 93:7 was retained.

The next stage of the synthesis of SynVesT-1 focused on nitro group reduction and in situ cyclization to access lactam **3** (Scheme 3). From the reactions screened, two methods were found to be suitable. The use of superstoichiometric amounts of iron in the presence of ammonium chloride gave lactam **3** in 62% yield.^9^ However, the use of nickel boride, formed in situ by the combination of nickel(II) chloride and sodium borohydride gave the best results. While others have used stoichiometric quantities of nickel(II) chloride for this transformation,^17^ we found that catalytic quantities (10 mol %) resulted in a fast, clean reaction at room temperature and gave lactam **3** in 63% yield.

The final step of the synthesis of SynVesT-1 required *N*-alkylation of lactam **3** with 4-(chloromethyl)-3-methylpyridine hydrochloride (**7**, Scheme 3). Although **7** is commercially available, it is expensive and thus, a scalable route, amenable for the multigram synthesis of this compound, was developed (Scheme 3). Using a procedure reported by Itoh and co-workers,^18^ the regioselective oxidation of 3,4-lutidine (**4**) was achieved using catalytic copper(II) chloride (10 mol %) and oxygen, which gave 3-methylisonicotinaldehyde (**5**) in 80% yield. Sodium borohydride reduction of aldehyde **5** and subsequent chlorination using thionyl chloride completed the three-step synthesis of **7** in 68% overall yield. *N*-Alkylation of lactam **3** with 4-(chloromethyl)-3-methylpyridine hydrochloride **7** using sodium hydride and catalytic tetrabutylammonium iodide (TBAI) gave SynVesT-1 in 73% yield. Analysis of the final SynVesT-1 compound by chiral HPLC showed that following nickel boride reduction and base-mediated alkylation, the enantiomeric ratio was retained (90:10).^15^

**Scheme 4:**
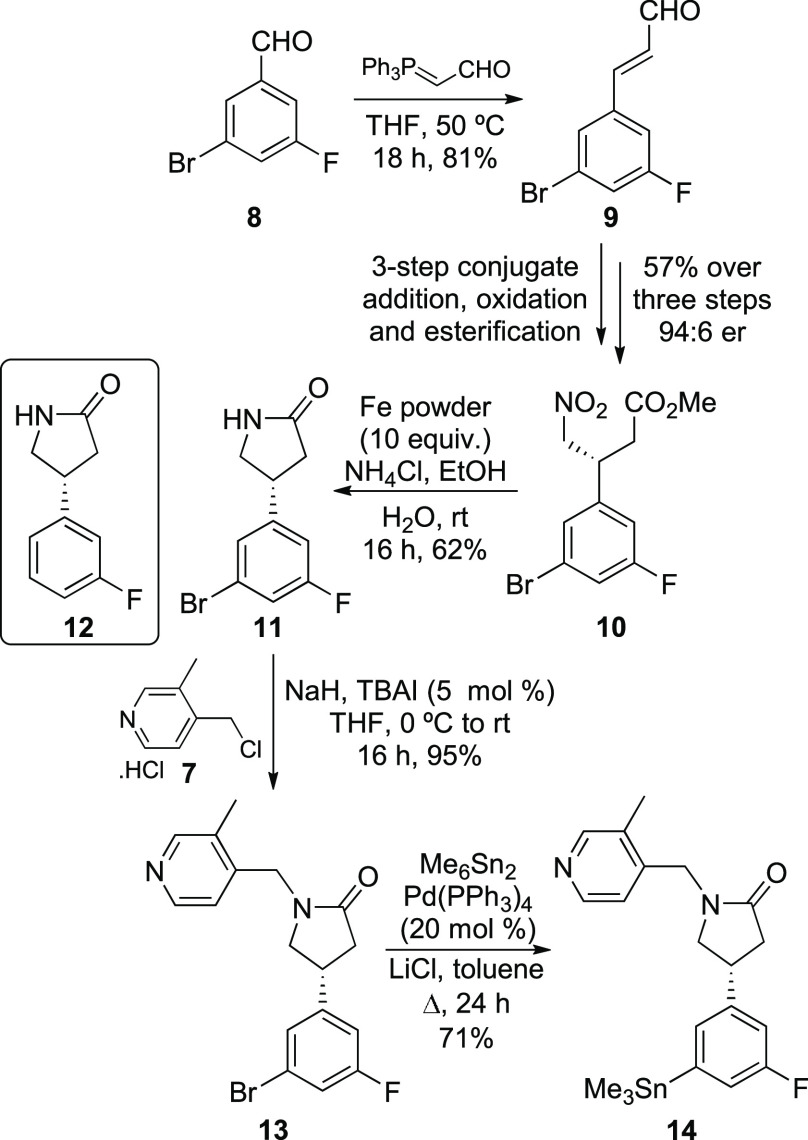
Asymmetric Synthesis of SynVesT-1 Organotin Precursor Isolated yields.

Having developed an asymmetric synthesis of nonradioactive SynVesT-1, the route was modified for the preparation of an organotin analogue that could be used for radiofluorination and the preparation of [^18^F]SynVesT-1. It was proposed that the substitution of one of the fluorine atoms for bromide would generate a late-stage intermediate that could be used in a palladium(0)-catalyzed stannylation reaction to access the organotin precursor. Therefore, the route was repeated using 3-bromo-5-fluorobenzaldehyde (**8**, Scheme 4). Wittig reaction with (triphenylphosphoranylidene)acetaldehyde gave *E*-cinnamaldehyde **9** in 81% yield. The optimized three-step, asymmetric conjugate addition reaction, oxidation, and esterification process, as described above gave 3-aryl-4-nitrobutanoate **10** in 57% overall yield and with an enantiomeric ratio of 94:6.^15^ To access the corresponding lactam, **11**, conditions for nitro group reduction that maintained the C–Br bond were investigated. While the use of nickel borate did generate **11** in 52% yield, it was isolated as an inseparable mixture with debrominated lactam **12** (9% yield). Instead, the use of iron powder and ammonium chloride for nitro group reduction and subsequent cyclization resulted in a chemoselective reaction, allowing the sole formation of lactam **11** in 62%. *N*-Alkylation of lactam **11** with 4-(chloromethyl)-3-methylpyridine hydrochloride **7**, under basic conditions and with catalytic TBAI gave **13** in 95% yield. Finally, palladium-catalyzed stannylation of **13** using hexamethylditin gave the organotin precursor in 71% yield.

**Scheme 5:**
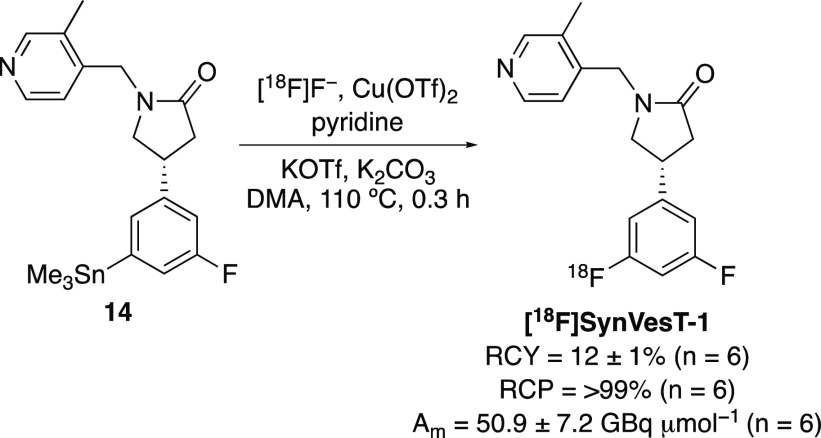
Synthesis of [^18^F]SynVesT-1

The radiosynthesis of [^18^F]SynVesT-1 using a TRACERlab FX_FN_ automated synthesizer and precursor **14** was next investigated. For these experiments, no-carrier-added [^18^F]fluoride from the cyclotron was trapped on a carbonate-preconditioned quaternary methyl ammonium (QMA) cartridge, eluted into the reactor, and then azeotropically dried. Radiofluorination of **14** was initially attempted using a previously reported copper(II) triflate mediated procedure,^8a^ however, this gave low radiochemical conversion (RCC, 28%) to [^18^F]SynVesT-1.^19^ Scott and co-workers recently demonstrated that low RCCs and protoarene byproduct formation during copper-mediated ^18^F-fluorinations of organoboron precursors could be overcome by considering the order of reagent addition.^20^ Using this strategy along with the late addition of **14**, copper(II) triflate, and pyridine to the synthesizer, followed by the introduction of [^18^F]fluoride, resulted in consistently higher RCCs. Further refinement of the procedure explored variation of reagent quantities, with a 20% reduction of Cu(OTf)_2_ leading to an optimal RCC of 72%. This optimized procedure was then used for the automated production, isolation, and purification of [^18^F]SynVesT-1 (Scheme 5). After a total synthesis and purification time of 57 min, this gave [^18^F]SynVesT-1 in a decay corrected radiochemical yield (RCY) of 12 ± 1% (*n* = 6) and with >99% radiochemical purity (RCP). The end of synthesis molar activity of [^18^F]SynVesT-1 using this procedure was found to be 50.9 ± 7.2 GBq μmol^–1^ starting from 58.5 ± 1.8 GBq of [^18^F]fluoride.

## Conclusions

In summary, a seven-step route has been developed for the asymmetric synthesis of SynVesT-1. The key steps involved a three-stage sequence of organocatalytic conjugate addition, Pinnick-type oxidation, and esterification to access an optically active 3-aryl-4-nitrobutanoate followed by a nickel borate-mediated reduction and cyclization to give a key lactam intermediate. The route was completed by *N*-alkylation of the lactam with 4-(chloromethyl)-3-methylpyridine hydrochloride for which, an inexpensive, scalable three-step synthesis was developed from 3,4-lutidine. This synthetic approach was also applicable for the asymmetric synthesis of an organotin analogue, which was used in an automated, copper(II)-mediated fluoro-destannylation for the preparation of [^18^F]SynVesT-1. By variation of the cinnamaldehyde and the final step *N*-alkylating agent, this synthetic strategy should be applicable for the stereoselective preparation of other heterocyclic nonacetamide PET ligands.

## Experimental Section

All reagents and starting materials were obtained from commercial sources and used as received unless otherwise stated. Dry solvents were purified using a PureSolv 500 MD solvent purification system. All reactions were performed under an atmosphere of air unless otherwise stated. Dry glassware was oven-dried at 140 °C for a minimum of 16 h, cooled to room temperature in vacuo, and then purged with argon. All reactions performed at elevated temperatures were heated using an oil bath. Brine is defined as a saturated aqueous solution of sodium chloride. Merck aluminum-backed plates precoated with silica gel 60 (UV_254_) were used for thin layer chromatography and were visualized under UV light (254/365 nm) then stained with iodine, potassium permanganate, vanillin, or ninhydrin solution. Flash column chromatography was carried out using Merck Geduran Si 60 (40–63 μm). ^1^H and ^13^C NMR spectra were recorded on Bruker DPX 400, Bruker AVI 400, Bruker AVIII 400 (^1^H 400 MHz; ^13^C 101 MHz) spectrometers or a Bruker AVIII 500 (^1^H 500 MHz; ^13^C 126 MHz) spectrometer with chemical shift values reported in ppm relative to tetramethylsilane (δ_H_ 0.00 and δ_C_ 0.0), CHCl_3_ (δ_H_ 7.26 and δ_C_ 77.2), or dimethyl sulfoxide (DMSO) (δ_H_ 2.50 and δ_C_ 39.5). Assignments of ^13^C NMR signals are based on distortionless enhancement by polarization transfer experiments. Mass spectra were obtained using a JEOL JMS-700 spectrometer or a Bruker microTOFq high resolution mass spectrometer. Melting points were determined on a Gallenkamp melting point apparatus and are uncorrected. Infrared spectra were recorded neat on a Shimadzu FTIR-84005 spectrometer. Optical rotations were determined as solutions irradiating with the sodium D line (λ = 598 nm) using an Autopol V polarimeter. [α]_D_ values are reported in units 10^–1^ deg. cm^2^ g^–1^. Chiral HPLC methods were calibrated with the corresponding racemic mixtures.

### (*E*)-3-(3′,5′-Difluorophenyl)prop-2-enal (**1**)

3,5-Difluorobenzaldehyde (3.00 g, 21.1 mmol) and (triphenylphosphoranylidene)acetaldehyde (7.06 g, 23.2 mmol) were dissolved in anhydrous THF (300 mL) under argon.^21^ The reaction mixture was heated to 50 °C and stirred for 21 h. The reaction mixture was cooled to room temperature and concentrated in vacuo. The resultant residue was dissolved in ethyl acetate (100 mL) and washed with water (100 mL). The aqueous layer was extracted with ethyl acetate (2 × 100 mL). The combined organic layers were dried (MgSO_4_), filtered, and concentrated in vacuo. Purification by flash column chromatography and eluting with 10% diethyl ether in petroleum ether (40–60) gave (*E*)-3-(3′,5′-difluorophenyl)prop-2-enal (**1**) as a pale yellow solid (2.63 g, 74%). Mp 94–96 °C; Spectroscopic data were consistent with previously published data.^21^^1^H NMR (500 MHz, CDCl_3_) δ 9.73 (d, 1H, *J* = 7.5 Hz,), 7.38 (d, 1H, *J* = 16.0 Hz), 7.11–7.05 (m, 2H), 6.89 (tt, 1H, *J* = 8.5, 2.3 Hz), 6.68 (dd, 1H, *J* = 16.0, 7.5 Hz); ^13^C{^1^H} NMR (126 MHz, CDCl_3_) δ 193.0 (CH), 163.4 (dd, 2 × C, ^1^*J*_CF_ = 250.2 Hz, ^3^*J*_CF_ = 12.6 Hz), 149.5 (t, CH, ^4^*J*_CF_ = 3.0 Hz), 137.3 (t, C, ^3^*J*_CF_ = 9.5 Hz), 130.7 (CH), 111.2 (dd, 2 × CH, ^2^*J*_CF_ = 19.8 Hz, ^4^*J*_CF_ = 6.3 Hz), 106.4 (t, CH, ^2^*J*_CF_ = 25.4 Hz); MS (ESI) *m/z* 191 (M + Na^+^, 100).

### Methyl (3*R*)-3-(3′,5′-difluorophenyl)-4-nitrobutanoate (**2**)

To a stirred solution of (*E*)-3-(3′,5′-difluorophenyl)prop-2-enal (**1**) (0.530 g, 3.15 mmol) in THF (8 mL) and water (1 mL) was added pivalic acid (0.0161 g, 0.158 mmol), boric acid (0.0977 g, 1.58 mmol), nitromethane (1.02 mL, 18.9 mmol), and (*R*)-α,α-bis[3,5-bis(trifluoromethyl)phenyl]-2-pyrrolidinemethanol trimethylsilyl ether (0.0944 g, 0.158 mmol). The reaction mixture was stirred at room temperature for 117 h and concentrated in vacuo. The resultant residue was dissolved in ethyl acetate (40 mL) and washed with 1 M aqueous hydrochloric acid (40 mL), saturated aqueous sodium bicarbonate (40 mL), and brine (40 mL). The organic layer was dried (MgSO_4_), filtered, and concentrated in vacuo to give (3*R*)-3-(3′,5′-difluorophenyl)-4-nitrobutanal as a yellow oil, which was used without further purification. (3*R*)-3-(3′,5′-Difluorophenyl)-4-nitrobutanal (0.722 g, 3.15 mmol) was dissolved in acetonitrile (16 mL) and cooled to 0 °C. To this was added a solution of potassium dihydrogen phosphate (0.320 g, 2.35 mmol) in water (8 mL), hydrogen peroxide (0.420 mL, 4.10 mmol, 30% w/w in water), and a solution of sodium chlorite (0.855 g, 9.45 mmol) in water (16 mL). The reaction mixture was stirred at 0 °C for 2 h and quenched with sodium sulfite (1.19 g, 9.45 mmol). The mixture was stirred at room temperature for 0.5 h. The reaction mixture was then acidified with 1 M aqueous potassium bisulfate (12 mL) and extracted with ethyl acetate (3 × 30 mL). The combined organic layers were dried (MgSO_4_), filtered, and concentrated in vacuo to give (3*R*)-3-(3′,5′-difluorophenyl)-4-nitrobutanoic acid as a yellow oil, which was used without further purification. To a stirred solution of (3*R*)-3-(3′,5′-difluorophenyl)-4-nitrobutanoic acid (0.772 g, 3.15 mmol) in methanol (11 mL) at 0 °C was added thionyl chloride (0.350 mL, 4.80 mmol). The reaction mixture was warmed to room temperature and then stirred under reflux for 1.5 h. The reaction mixture was cooled to room temperature and concentrated in vacuo. The resultant residue was dissolved in ethyl acetate (40 mL) and washed with water (40 mL). The aqueous layer was extracted with ethyl acetate (2 × 40 mL). The combined organic layers were dried (MgSO_4_), filtered, and concentrated in vacuo. Purification by flash column chromatography and eluting with 60% dichloromethane in hexane gave methyl (3*R*)-3-(3′,5′-difluorophenyl)-4-nitrobutanoate (**2**) as a colorless oil, which solidified upon standing (0.449 g, 55% over three steps). Mp 51–53 °C; IR (neat) 2959, 1732, 1624, 1597, 1551, 1439, 1373, 1119, 980, 856 cm^–1^; [α]_D_^19^ +9.6 (*c* 0.1, CHCl_3_); ^1^H NMR (400 MHz, CDCl_3_) δ 6.82–6.75 (m, 2H), 6.74 (tt, 1H, *J* = 8.6, 2.2 Hz), 4.73 (dd, 1H, *J* = 13.0, 6.6 Hz), 4.62 (dd, 1H, *J* = 13.0, 8.2 Hz), 4.03–3.93 (m, 1H), 3.67 (s, 3H), 2.75 (dd, 2H, *J* = 7.4, 1.8 Hz); ^13^C{^1^H} NMR (101 MHz, CDCl_3_) δ 170.6 (C), 163.4 (dd, 2 × CH, ^1^*J*_CF_ = 251.1 Hz, ^3^*J*_CF_ = 12.9 Hz), 142.3 (t, C, ^3^*J*_CF_ = 8.9 Hz), 110.7 (dd, 2 × CH, ^2^*J*_CF_ = 18.8 Hz, ^4^*J*_CF_ = 7.3 Hz), 103.9 (t, CH, ^2^*J*_CF_ = 25.3 Hz), 78.8 (CH_2_), 52.3 (CH_3_), 39.8 (t, CH, ^4^*J*_CF_ = 2.1 Hz), 37.2 (CH_2_); MS (ESI) *m/z* 282 (M + Na^+^, 100); HRMS (ESI) *m/z*: [M + Na]^+^ calcd for C_11_H_11_F_2_NNaO_4_ 282.0548; found 282.0549. HPLC (AD-H, *i*-propanol/*n*-hexane = 5/95, flow rate = 1.0 mL/min, λ = 254 nm) *t*_R_ = 12.3 min (minor), 14.8 min (major), 93:7 er.

### (4*R*)-4-(3′,5′-Difluorophenyl)pyrrolidin-2-one (**3**)

To a stirred solution of methyl (3*R*)-3-(3′,5′-difluorophenyl)-4-nitrobutanoate (**2**) (0.418 g, 1.61 mmol) in methanol (8 mL) was added nickel(II) chloride hexahydrate (0.0383 g, 0.161 mmol). Sodium borohydride (0.305 g, 8.05 mmol) was added in four portions over 0.2 h, producing a black precipitate and the evolution of gas.^10^ The reaction mixture was stirred at room temperature for 0.75 h and then filtered through a short pad of Celite with dichloromethane (30 mL). The filtrate was concentrated in vacuo. The resultant residue was dissolved in chloroform (20 mL) and washed with water (20 mL). The organic and aqueous layers were separated, and the aqueous layer was extracted with chloroform (2 × 20 mL). The combined organic layers were dried (MgSO_4_), filtered, and concentrated in vacuo. Purification by flash column chromatography and eluting with 5% methanol in diethyl ether gave (4*R*)-4-(3′,5′-difluorophenyl)pyrrolidin-2-one (**3**) as a white solid (0.202 g, 63%). Mp 94–96 °C; [α]_D_^19^–25.9 (*c* 0.1, CHCl_3_); Spectroscopic data were consistent with previously published data.^10^^1^H NMR (400 MHz, CDCl_3_) δ 6.81–6.67 (m, 4H), 3.83–3.75 (m, 1H), 3.72–3.61 (m, 1H), 3.39 (dd, 1H, *J* = 9.6, 6.8 Hz), 2.74 (dd, 1H, *J* = 16.9, 9.0 Hz), 2.44 (dd, 1H, *J* = 16.9, 8.2 Hz); ^13^C{^1^H} NMR (101 MHz, CDCl_3_) δ 177.1 (C), 163.4 (dd, 2 × C, ^1^*J*_CF_ = 250.1 Hz, ^3^*J*_CF_ = 12.9 Hz), 146.2 (t, C, ^3^*J*_CF_ = 8.8 Hz), 109.9 (dd, 2 × CH, ^2^*J*_CF_ = 18.5 Hz, ^4^*J*_CF_ = 7.0 Hz), 102.8 (t, CH, ^2^*J*_CF_ = 25.2 Hz), 49.1 (CH_2_), 40.0 (t, CH, ^4^*J*_CF_ = 2.1 Hz), 37.7 (CH_2_); MS (ESI) *m/z* 220 (M + Na^+^, 100). HPLC (AD-H, *i*-propanol/*n*-hexane = 5/95, flow rate = 1.0 mL/min, λ = 254 nm) *t*_R_ = 18.8 min (major), 26.1 min (minor), 94:6 er.

### (4*R*)-4-(3′,5′-Difluorophenyl)-1-[(3″-methylpyridin-4″-yl)methyl]pyrrolidin-2-one (SynVesT-1)

Sodium hydride (20.0 mg, 0.469 mmol, 60% dispersion in mineral oil) was added to an oven-dried flask under argon and cooled to 0 °C.^8b^ This was washed with hexane (0.5 mL) and then dried in vacuo at room temperature for 0.5 h. The resultant sodium hydride was then suspended in anhydrous THF (0.5 mL) and cooled to 0 °C. To the flask containing the sodium hydride suspension was added a solution of (4*R*)-4-(3′,5′-difluorophenyl)pyrrolidin-2-one (**3**) (42.0 mg, 0.213 mmol) in anhydrous THF (0.5 mL). The reaction mixture was stirred for 0.5 h at 0 °C. TBAI (4.00 mg, 0.0110 mmol) was then added followed by 4-(chloromethyl)-3-methylpyridine hydrochloride (**7**) (42.0 mg, 0.234 mmol). The resultant solution was warmed to room temperature and stirred for 16 h. After cooling to 0 °C, the reaction was quenched with saturated aqueous sodium bicarbonate (2.5 mL) and the aqueous solution was extracted with chloroform (3 × 5 mL). The combined organic layers were dried (MgSO_4_), filtered, and concentrated in vacuo. Purification by flash column chromatography and eluting with a gradient of 5–7.5% methanol in diethyl ether gave (4*R*)-4-(3′,5′-difluorophenyl)-1-[(3″-methylpyridin-4″-yl)methyl]pyrrolidin-2-one (SynVesT-1) as a white solid (0.0470 g, 73%). Mp 106–108 °C; [α]_D_^19^ +21.9 (*c* 0.1, CHCl_3_); Spectroscopic data were consistent with previously published data.^8b^^1^H NMR (400 MHz, CDCl_3_) δ 8.45–8.39 (m, 2H), 7.04 (d, 1H, *J* = 5.2 Hz), 6.76–6.66 (m, 3H), 4.62 (d, 1H, *J* = 15.6 Hz), 4.42 (d, 1H, *J* = 15.6 Hz), 3.66–3.53 (m, 2H), 3.24 (dd, 1H, *J* = 8.0, 5.6 Hz), 2.91 (dd, 1H, *J* = 17.0, 8.6 Hz), 2.60 (dd, 1H, *J* = 17.0, 8.2 Hz), 2.31 (s, 3H); ^13^C{^1^H} NMR (101 MHz, CDCl_3_) δ 173.2 (C), 163.4 (dd, 2 × C, ^1^*J*_CF_ = 250.4 Hz, ^3^*J*_CF_ = 12.8 Hz), 151.5 (CH), 148.1 (CH), 145.8 (t, C, ^3^*J*_CF_ = 8.8 Hz), 142.8 (C), 131.7 (C), 122.5 (CH), 109.8 (dd, 2 × CH, ^2^*J*_CF_ = 18.6 Hz, ^4^*J*_CF_ = 7.0 Hz), 102.9 (t, CH, ^2^*J*_CF_ = 25.4 Hz), 53.5 (CH_2_), 43.7 (CH_2_), 38.2 (CH_2_), 37.1 (t, CH, ^4^*J*_CF_ = 2.1 Hz), 16.1 (CH_3_); MS (ESI) *m/z* 303 (M + H^+^, 100). HPLC (AD-H, *i*-propanol/*n*-hexane = 10/90, flow rate = 2.0 mL/min, λ = 254 nm) *t*_R_ = 15.8 min (minor), 19.5 min (major), 90:10 er.

### 3-Methylisonicotinaldehyde (**5**)

3,4-Lutidine (**4**) (5.00 g, 46.7 mmol) and copper(II) chloride dihydrate (0.796 g, 4.67 mmol) were dissolved in dimethyl sulfoxide (140 mL).^22^ Oxygen was then bubbled through the solution using a balloon for 0.75 h. A fresh oxygen balloon was used to maintain the oxygen atmosphere throughout the reaction. The solution was then heated to 100 °C and stirred for 24 h. The reaction mixture was diluted with dichloromethane (100 mL) and washed with saturated sodium bicarbonate solution (100 mL) The aqueous layer was extracted with dichloromethane (3 × 100 mL). The combined organic layers were then dried (MgSO_4_), filtered, and concentrated under reduced pressure. Purification by flash column chromatography and eluting with 50% ethyl acetate in hexane gave 3-methylisonicotinaldehyde (**5**) (3.05 g, 80%) as a brown oil. Spectroscopic data were consistent with previously published data.^22^^1^H NMR (500 MHz, CDCl_3_) δ 10.29 (s, 1H), 8.67 (d, 1H, *J* = 5.3 Hz), 8.60 (s, 1H), 7.57 (d, 1H, *J* = 5.3 Hz), 2.61 (s, 3H); ^13^C{^1^H} NMR (126 MHz, CDCl_3_) δ 192.2 (CH), 153.3 (CH), 148.8 (CH), 139.1 (C), 133.0 (C), 123.2 (CH), 16.3 (CH_3_); MS (EI) *m*/*z* 121 (M^+^, 100).

### (3-Methylpyridin-4-yl)methanol (**6**)

3-Methylisonicotinaldehyde (**5**) (3.00 g, 24.8 mmol) was dissolved in anhydrous methanol under an argon atmosphere and cooled to 0 °C.^23^ To this solution, sodium borohydride (1.03 g, 27.2 mmol) was added portionwise. Once gas evolution had ceased, the solution was then allowed to warm to room temperature and stirred for 21 h. The reaction was quenched by the addition of saturated ammonium chloride solution (15 mL) and all volatiles were removed in vacuo. The resulting residue was dissolved in a mixture of ethyl acetate (50 mL) and water (50 mL). The organic and aqueous layers were separated and the aqueous layer was extracted with ethyl acetate (5 × 50 mL). The combined organic layers were then dried (MgSO_4_) and filtered. Concentration under vacuum gave (3-methylpyridin-4-yl)methanol (**6**) (2.70 g, 89%) as a white solid. Mp 77–78 °C (lit.^23^ 81–82 °C); ^1^H NMR (500 MHz, DMSO-d_6_) δ 8.37 (d, 1H, *J* = 5.0 Hz), 8.28 (s, 1H), 7.38 (d, 1H, *J* = 5.0 Hz), 5.39 (br s, 1H), 4.51 (s, 2H), 2.17 (s, 3H); ^13^C{^1^H} NMR (126 MHz, DMSO-d_6_) δ 149.5 (C), 149.4 (CH), 147.3 (CH), 129.9 (C), 120.2 (CH), 59.6 (CH_2_), 14.9 (CH_3_); MS (EI) *m/z* 123 (M^+^, 100).

### 4-(Chloromethyl)-3-methylpyridine Hydrochloride (**7**)

(3-Methylpyridin-4-yl)methanol (**6**) (2.60 g, 21.1 mmol) was dissolved in dichloromethane (30 mL) under an argon atmosphere. The resulting solution was cooled to 0 °C and thionyl chloride (10.1 g, 84.4 mmol) was added slowly. Once the addition was complete, the solution was warmed to room temperature and stirred for 18 h. Upon completion of the reaction, removal of all volatiles in vacuo gave 4-(chloromethyl)-3-methylpyridine hydrochloride (**7**) (3.61 g, 96%) as an off-white solid. Mp 177–179 °C; ^1^H NMR (400 MHz, DMSO-d_6_) δ 8.83 (s, 1H), 8.79 (d, 1H, *J* = 6.0 Hz), 8.05 (d, 1H, *J* = 6.0 Hz), 5.05 (s, 2H), 2.49 (s, 3H); ^13^C{^1^H} NMR (101 MHz, DMSO-d_6_) δ 154.0 (C), 142.1 (CH), 140.2 (CH), 136.5 (C), 126.0 (CH), 41.9 (CH_2_), 15.3 (CH_3_); MS (ESI) 142 (M + H^+^, 100); HRMS (ESI) *m/z*: [M + H]^+^ calcd for C_7_H_9_^35^ClN 142.0418; found 142.0419.

### (*E*)-3-(3′-Bromo-5′-fluorophenyl)prop-2-enal (**9**)

3-Bromo-5-fluorobenzaldehyde (**8**) (6.09 g, 30.0 mmol) and (triphenylphosphoranylidene)acetaldehyde (10.0 g, 33.0 mmol) were dissolved in anhydrous THF (300 mL) under argon. The reaction mixture was heated to 50 °C and stirred for 18 h. The reaction mixture was cooled to room temperature and concentrated in vacuo. Purification by flash column chromatography and eluting with 10% diethyl ether in hexane gave (*E*)-3-(3′-bromo-5′-fluorophenyl)prop-2-enal (**9**) as a pale yellow solid (5.57 g, 81%). Mp 65–67 °C; IR (neat) 3071, 2862, 1659, 1628, 1574, 1427, 1269, 1126, 976, 849 cm^–1^; ^1^H NMR (400 MHz, CDCl_3_) δ 9.72 (d, 1H, *J* = 7.6 Hz), 7.51–7.48 (m, 1H), 7.36 (d, 1H, *J* = 16.0 Hz), 7.32 (dt, 1H, *J* = 7.8, 2.0 Hz), 7.20 (dt, 1H, *J* = 8.9, 2.0 Hz), 6.68 (dd, 1H, *J* = 16.0, 7.6 Hz); ^13^C{^1^H} NMR (101 MHz, CDCl_3_) δ 192.9 (CH), 162.9 (d, C, ^1^*J*_CF_ = 253.2 Hz), 149.1 (d, CH, ^4^*J*_CF_ = 2.7 Hz), 137.6 (d, C, ^3^*J*_CF_ = 8.3 Hz), 130.8 (CH), 127.5 (d, CH, ^4^*J*_CF_ = 3.2 Hz), 123.5 (d, C, ^3^*J*_CF_ = 9.9 Hz), 121.5 (d, CH, ^2^*J*_CF_ = 24.6 Hz), 113.8 (d, CH, ^2^*J*_CF_ = 22.2 Hz); MS (ESI) *m/z* 251 (M + Na^+^, 100); HRMS (ESI) *m/z*: [M + Na]^+^ calcd for C_9_H_6_^79^BrFNaO 250.9478; found 250.9469.

### Methyl (3*R*)-3-(3′-bromo-5′-fluorophenyl)-4-nitrobutanoate (**10**)

To a stirred solution of (*E*)-3-(3′-bromo-5′-fluorophenyl)prop-2-enal (**9**) (0.23 g, 1.0 mmol) in THF (3 mL) and water (0.5 mL) was added pivalic acid (0.0051 g, 0.050 mmol), boric acid (0.031 g, 0.50 mmol), nitromethane (0.33 mL, 6.0 mmol), and (*R*)-α,α-bis[3,5-bis(trifluoromethyl)phenyl]-2-pyrrolidinemethanol trimethylsilyl ether (0.030 g, 0.050 mmol). The reaction mixture was stirred at room temperature for 117 h and concentrated in vacuo. The resultant residue was dissolved in ethyl acetate (20 mL) and washed with 1 M aqueous hydrochloric acid (20 mL), saturated aqueous sodium bicarbonate (20 mL), and brine (20 mL). The organic layer was dried (MgSO_4_), filtered, and concentrated in vacuo to give (3*R*)-3-(3′-bromo-5′-fluorophenyl)-4-nitrobutanal as a yellow oil, which was used without further purification. (3*R*)-3-(3′-Bromo-5′-fluorophenyl)-4-nitrobutanal (0.290 g, 1.00 mmol) was dissolved in acetonitrile (5 mL) and cooled to 0 °C. To this was added a solution of potassium dihydrogen phosphate (0.104 g, 0.764 mmol) in water (2.5 mL), hydrogen peroxide (0.140 mL, 1.37 mmol, 30% w/w in water), and a solution of sodium chlorite (0.271 g, 3.00 mmol) in water (5 mL). The reaction mixture was stirred at 0 °C for 3 h and quenched with sodium sulfite (0.378 g, 3.00 mmol). The mixture was stirred at room temperature for 0.5 h. The reaction mixture was then acidified with 1 M aqueous potassium bisulfate (4 mL) and extracted with ethyl acetate (3 × 15 mL). The combined organic layers were dried (MgSO_4_), filtered, and concentrated in vacuo to give (3*R*)-3-(3′-bromo-5′-fluorophenyl)-4-nitrobutanoic acid as a yellow oil, which was used without further purification. To a stirred solution of (3*R*)-3-(3′-bromo-5′-fluorophenyl)-4-nitrobutanoic acid (0.306 g, 1.00 mmol) in methanol (3 mL) at 0 °C was added thionyl chloride (0.110 mL, 1.50 mmol). The reaction mixture was warmed to room temperature and then stirred under reflux for 1.5 h. The reaction mixture was cooled to room temperature and concentrated in vacuo. The resultant residue was dissolved in ethyl acetate (20 mL) and washed with water (20 mL). The aqueous layer was extracted with ethyl acetate (2 × 20 mL). The combined organic layers were dried (MgSO_4_), filtered, and concentrated in vacuo. Purification by flash column chromatography and eluting with 60% dichloromethane in hexane gave methyl (3*R*)-3-(3′-bromo-5′-fluorophenyl)-4-nitrobutanoate (**10**) as a colorless oil, which solidified upon standing (0.181 g, 57% over three steps). Mp 39–40 °C; IR (neat) 3082, 2955, 1724, 1547, 1435, 1373, 1354, 1269, 1219, 1173, 914, 864 cm^–1^; [α]_D_^19^ +7.6 (*c* 0.1, CHCl_3_); ^1^H NMR (400 MHz, CDCl_3_) δ 7.21–7.15 (m, 2H), 6.91 (dt, 1H, *J* = 8.9, 1.9 Hz), 4.72 (dd, 1H, *J* = 13.2, 6.4 Hz), 4.61 (dd, 1H, *J* = 13.2, 8.2 Hz), 4.00–3.91 (m, 1H), 3.66 (s, 3H), 2.74 (dd, 2H, *J* = 7.4, 2.2 Hz); ^13^C{^1^H} NMR (101 MHz, CDCl_3_) δ 170.6 (C), 162.8 (d, C, ^1^*J*_CF_ = 253.2 Hz), 142.5 (d, C, ^3^*J*_CF_ = 7.7 Hz), 126.6 (d, CH, ^4^*J*_CF_ = 3.2 Hz), 123.4 (d, C, ^3^*J*_CF_ = 9.9 Hz), 119.1 (d, CH, ^2^*J*_CF_ = 24.3 Hz), 113.8 (d, CH, ^2^*J*_CF_ = 22.1 Hz), 78.7 (CH_2_), 52.3 (CH_3_), 39.6 (d, CH, ^4^*J*_CF_ = 1.8 Hz), 37.2 (CH_2_); MS (ESI) *m/z* 342 (M + Na^+^, 100); HRMS (ESI) *m/z*: [M + Na]^+^ calcd for C_11_H_11_^79^BrFNNaO_4_ 341.9748; found 341.9742. HPLC (AD-H, *i*-propanol/*n*-hexane = 2/98, flow rate = 1.0 mL/min, λ = 254 nm) *t*_R_ = 20.3 min (minor), 22.35 min (major), 94:6 er.

### (4*R*)-4-(3′-Bromo-5′-fluorophenyl)pyrrolidin-2-one (**11**)

To a stirred solution of methyl (3*R*)-3-(3′-bromo-5′-fluorophenyl)-4-nitrobutanoate (**10**) (200 mg, 0.625 mmol) in ethanol (4 mL), methanol (2 mL), and water (2 mL) was added iron powder (349 mg, 6.25 mmol) and ammonium chloride (1.14 g, 21.3 mmol).^10^ The reaction mixture was stirred at room temperature for 16 h and then adjusted to pH ≥ 11 with 6 M aqueous solution of sodium hydroxide (4 mL). The reaction mixture was stirred at room temperature for 0.5 h and then filtered through a short pad of Celite with chloroform (75 mL). The filtrate was concentrated in vacuo. The resultant residue was dissolved in chloroform (40 mL) and washed with water (40 mL). The organic and aqueous layers were separated, and the aqueous layer was extracted with chloroform (2 × 40 mL). The combined organic layers were dried (MgSO_4_), filtered, and concentrated in vacuo. Purification by flash column chromatography and eluting with 5% methanol in diethyl ether gave (4*R*)-4-(3′-bromo-5′-fluorophenyl)pyrrolidin-2-one (**11**) as a white solid (99.7 mg, 62%). Mp 110–111 °C; [α]_D_^20^–18.5 (*c* 0.1, CHCl_3_). Spectroscopic data were consistent with previously published data.^10^^1^H NMR (400 MHz, CDCl_3_) δ 7.21–7.18 (m, 1H), 7.16 (dt, 1H, *J* = 8.0, 2.0 Hz), 6.92 (dt, 1H, *J* = 9.4, 2.0 Hz), 6.42 (br s, 1H), 3.83–3.75 (m, 1H), 3.72–3.60 (m, 1H), 3.39 (dd, 1H, *J* = 9.6, 6.8 Hz), 2.74 (dd, 1H, *J* = 17.0, 9.0 Hz), 2.44 (dd, 1H, *J* = 17.0, 8.2 Hz); ^13^C{^1^H} NMR (101 MHz, CDCl_3_) δ 177.0 (C), 163.0 (d, C, ^1^*J*_CF_ = 252.5 Hz), 146.4 (d, C, ^3^*J*_CF_ = 7.7 Hz), 126.0 (d, CH, ^4^*J*_CF_ = 3.1 Hz), 123.2 (d, C, ^3^*J*_CF_ = 10.0 Hz), 118.1 (d, CH, ^2^*J*_CF_ = 24.5 Hz), 113.0 (d, CH, ^2^*J*_CF_ = 21.8 Hz), 49.1 (CH_2_), 39.8 (d, CH, ^4^*J*_CF_ = 1.8 Hz), 37.7 (CH_2_); MS (ESI) *m/z* 280 (M + Na^+^, 100).

### (4*R*)-4-(3′-Bromo-5′-fluorophenyl)-1-[(3″-methylpyridin-4″-yl)methyl]pyrrolidin-2-one (**13**)

Sodium hydride (53.0 mg, 1.32 mmol, 60% dispersion in mineral oil) was added to an oven-dried flask under argon and cooled to 0 °C.^8b^ This was washed with hexane (1 mL) and then dried in vacuo at room temperature for 0.5 h. The resultant sodium hydride was then suspended in anhydrous THF (1.5 mL) and cooled to 0 °C. To the flask containing the sodium hydride suspension was added a solution of (4*R*)-4-(3′-bromo-5′-fluorophenyl)pyrrolidin-2-one (**11**) (155 mg, 0.600 mmol) in anhydrous THF (1.5 mL). The reaction mixture was stirred for 0.5 h at 0 °C. TBAI (11.1 mg, 0.0300 mmol) was then added followed by 4-(chloromethyl)-3-methylpyridine hydrochloride (**7**) (118 mg, 0.660 mmol). The resultant solution was warmed to room temperature and stirred for 16 h. After cooling to 0 °C, the reaction was quenched with saturated aqueous sodium bicarbonate (5 mL) and the aqueous solution was extracted with chloroform (3 × 5 mL). The combined organic layers were dried (MgSO_4_), filtered, and concentrated in vacuo. Purification by flash column chromatography and eluting with a gradient of 5–7.5% methanol in diethyl ether gave (4*R*)-4-(3′-bromo-5′-fluorophenyl)-1-[(3″-methylpyridin-4″-yl)methyl]pyrrolidin-2-one (**13**) as a pale yellow solid (207 mg, 95%). Mp 113–115 °C; [α]_D_^18^ +28.9 (*c* 0.1, CHCl_3_). Spectroscopic data were consistent with previously published data.^8b^^1^H NMR (400 MHz, CDCl_3_) δ 8.46–8.38 (m, 2H), 7.15 (dt, 1H, *J* = 8.0, 2.0 Hz), 7.11 (br t, 1H, *J* = 2.0 Hz), 7.04 (d, 1H, *J* = 4.8 Hz), 6.84 (dt, 1H, *J* = 9.2, 2.0 Hz), 4.59 (d, 1H, *J* = 15.4 Hz), 4.43 (d, 1H, *J* = 15.4 Hz), 3.66–3.51 (m, 2H), 3.23 (dd, 1H, *J* = 9.2, 6.0 Hz), 2.91 (dd, 1H, *J* = 17.0, 8.6 Hz), 2.59 (dd, 1H, *J* = 17.0, 7.8 Hz), 2.31 (s, 3H); ^13^C{^1^H} NMR (101 MHz, CDCl_3_) δ 173.1 (C), 162.9 (d, C, ^1^*J*_CF_ = 252.8 Hz), 151.6 (CH), 148.2 (CH), 146.0 (d, C, ^3^*J*_CF_ = 7.6 Hz), 142.7 (C), 131.7 (C), 125.9 (d, CH, ^4^*J*_CF_ = 3.2 Hz), 123.3 (d, C, ^3^*J*_CF_ = 10.0 Hz), 122.6 (CH), 118.2 (d, CH, ^2^*J*_CF_ = 24.4 Hz), 113.0 (d, CH, ^2^*J*_CF_ = 21.8 Hz), 53.5 (CH_2_), 43.7 (CH_2_), 38.2 (CH_2_), 36.9 (d, CH, ^4^*J*_CF_ = 1.9 Hz), 16.1 (CH_3_); MS (ESI) *m/z* 363 (M + H^+^, 100).

### (4*R*)-4-[3′-Fluoro-5′-(trimethylstannyl)phenyl]-1-[(3″-methylpyridin-4″-yl)methyl]pyrrolidin-2-one (**14**)

Lithium chloride (0.265 g, 6.25 mmol) was added to a flask and dried in an oven at 140 °C overnight. (4*R*)-4-(3′-bromo-5′-fluorophenyl)-1-[(3″-methylpyridin-4″-yl)methyl]pyrrolidin-2-one (**13**) (0.454 g, 1.25 mmol) was dried under high vacuum for 1 h, purged with argon, and dissolved in anhydrous toluene (13 mL).^8b^ The oven-dried flask was cooled to room temperature in vacuo and then purged with argon. The (4*R*)-4-(3′-bromo-5′-fluorophenyl)-1-[(3″-methylpyridin-4″-yl)methyl]pyrrolidin-2-one (**13**) solution was added to the flask and degassed under argon for 0.33 h. Tetrakis(triphenylphosphine)palladium(0) (0.289 g, 0.250 mmol) was added and the mixture degassed under argon for further 0.2 h. Hexamethylditin (0.520 mL, 2.51 mmol) was added and the reaction mixture was stirred under reflux for 24 h. After cooling to room temperature, the reaction was quenched with aqueous potassium fluoride (3 mL, 30% w/w) and stirred for 1 h. The crude mixture was filtered through a short pad of Celite with ethyl acetate (300 mL) and concentrated in vacuo. Purification by flash column chromatography and eluting with 5% methanol in diethyl ether gave (4*R*)-4-[3′-fluoro-5′-(trimethylstannyl)phenyl]-1-[(3″-methylpyridin-4″-yl)methyl] pyrrolidin-2-one (**14**) as a white solid (0.400 g, 71%). Mp 106–108 °C; [α]_D_^20^ +25.1 (*c* 0.1, CHCl_3_). Spectroscopic data were consistent with previously published data.^8b^^1^H NMR (400 MHz, CDCl_3_) δ 8.44–8.39 (m, 2H), 7.11–7.01 (m, 3H), 6.82 (dt, 1H, *J* = 10.0, 2.0 Hz), 4.63 (d, 1H, *J* = 15.6 Hz), 4.41 (d, 1H, *J* = 15.6 Hz), 3.67–3.54 (m, 2H), 3.33–3.21 (m, 1H), 2.92 (dd, 1H, *J* = 17.0, 9.0 Hz), 2.65 (dd, 1H, *J* = 17.0, 8.2 Hz), 2.31 (s, 3H), 0.29 (s, 9H); ^13^C{^1^H} NMR (101 MHz, CDCl_3_) δ 173.8 (C), 162.9 (d, C, ^1^*J*_CF_ = 252.8 Hz), 151.4 (CH), 148.1 (CH), 146.4 (d, C, ^3^*J*_CF_ = 2.8 Hz), 143.8 (d, C, ^3^*J*_CF_ = 5.6 Hz), 143.0 (C), 131.7 (C), 129.7 (d, CH, ^4^*J*_CF_ = 2.8 Hz), 122.4 (CH), 121.0 (d, CH, ^2^*J*_CF_ = 17.5 Hz), 113.4 (d, CH, ^2^*J*_CF_ = 21.8 Hz), 54.1 (CH_2_), 43.7 (CH_2_), 38.5 (CH_2_), 37.1 (d, CH, ^4^*J*_CF_ = 1.6 Hz), 16.1 (CH_3_), −9.3 (3 × CH_3_); MS (ESI) *m/z* 449 (M + H^+^, 100).

### Radiochemistry: General Experimental Method

No-carrier-added aqueous [^18^F]fluoride was produced via the ^18^O(p,n)^18^F nuclear reaction by irradiation of ^18^O-enriched water by a GE PETtrace 8 cyclotron. All radiofluorination reactions were carried out on a GE TRACERlab FXFN automated synthesizer. Sep-Pak QMA Carbonate Plus Light cartridges (Waters) were preconditioned with water (10 mL) prior to use. Oasis HLB Plus Light (Waters) cartridges were preconditioned with ethanol (5 mL) and then with water (10 mL) prior to use. The starting activity for calculating the RCY was determined from the Geiger–Müller (GM) reading taken immediately following the delivery of [^18^F]fluoride to the synthesizer from the cyclotron. The final activity readings were recorded using a Capintec CRC-25 PET dose calibrator. Analytical HPLC was carried out on a Thermo Dionex Ulimate system 3000 equipped with a Berthold FlowStar LB 513 radio flow detector and a DAD-3000 UV detector. An isocratic mobile phase of 60% acetonitrile in water was used with an Agilent Pursuit XRs 5 C18 250 mm × 4.0 mm column at a rate of 1 mL min^–1^. The nonradioactive standards were detected using a UV wavelength of 267 nm.

### **[^18^F]SynVesT-1**

Immediately prior to delivering [^18^F]fluoride, (4*R*)-4-[3′-fluoro-5′-(trimethylstannyl)phenyl]-1-[(3″-methylpyridin-4″-yl)methyl] pyrrolidin-2-one (**14**) (5.0 mg), copper(II) trifluoromethanesulfonate (8.0 mg), and pyridine (20 μL) were dissolved in *N*,*N*-dimethylacetamide (0.7 mL) and the solution was added to a vial on the synthesizer. Cyclotron target water containing [^18^F]fluoride was transferred to and trapped on a Sep-Pak QMA Carbonate Plus Light 46 mg cartridge. The activity was eluted into a reaction vessel using a solution of potassium trifluoromethanesulfonate (10 mg) and potassium carbonate (2.4 μg) in water (0.55 mL). This solution was dried by stirring at 100 °C under vacuum and a stream of helium gas for 2 min. This process was repeated twice using acetonitrile (2 × 1 mL). The [^18^F]fluoride was then completely dried by applying full vacuum for 1 min. The solution of (4*R*)-4-[3′-fluoro-5′-(trimethylstannyl)phenyl]-1-[(3″-methylpyridin-4″-yl)methyl] pyrrolidin-2-one (**14**) was added to the reaction vessel, which was sealed, and the mixture was heated to 110 °C for 20 min while being stirred. The reaction mixture was cooled to 30 °C and diluted with a 50% aqueous solution of acetonitrile (2.0 mL). The reaction mixture was then transferred into the HPLC injector loop for purification. Purification was performed by semipreparative HPLC with a SYKMN S1122 solvent delivery system using a Phenomenex Luna 5 μm C18 100 Å, 250 mm × 10 mm column and eluted using a 50% solution of acetonitrile in aqueous 0.02 M ammonium acetate at a flow rate of 4 mL min^–1^. The product fraction was identified using a gamma detector at a retention time of approximately 12 min and collected into a flask containing water (20 mL). The diluted fraction was then passed onto an Oasis HLB Plus Light cartridge, washed with water (10 mL), and eluted from the cartridge with ethanol (1.0 mL) and saline (9.0 mL). [^18^F]SynVesT-1 was isolated in 12 ± 1% RCY with a RCP of >99% and a molar activity of 50.9 ± 7.2 GBq μmol^–1^, starting from 58.5 ± 1.8 GBq of [^18^F]fluoride (*n* = 6). The total synthesis time from the delivery of [^18^F]fluoride to the extraction of the product was 57 min.

## Notes

The data underlying this study are available in the published article and its online supplementary material.

## Supporting Information Available

Chiral and racemic HPLC traces for compounds **2**, **3**, SynVesT-1, and **10**; HPLC traces for SynVesT-1 and [^18^F]SynVesT-1; ^1^H and ^13^C NMR spectra of all compounds (PDF)
