# Supplementary material for: Organocatalytic Asymmetric Synthesis of SynVesT-1, a Synaptic Density Positron Emission Tomography Imaging Agent
Source: J Org Chem. 2022 Oct 12;87(21):14443–51. doi: 10.1021/acs.joc.2c01895 (PMC9639009; doi:10.1021/acs.joc.2c01895)

Supporting Information for:

Organocatalytic Asymmetric Synthesis of SynVesT-1, a Synaptic Density Positron Emission Tomography Imaging Agent

Holly McErlain,[†] Euan B. McLean,[†] Timaeus E. F. Morgan,[‡] Valeria K. Burianova,[†]

Adriana A. S. Tavares[‡] and Andrew Sutherland*[†]

[†]School of Chemistry, The Joseph Black Building, University of Glasgow, Glasgow G12 8QQ, United Kingdom. [‡]BHF-University Centre for Cardiovascular Science, University of Edinburgh, Edinburgh EH16 4TJ, United Kingdom.

Table of Contents

1. Chiral and Racemic HPLC Traces for compounds **2**, **3**, **SynVesT-1** and **10** S2–S9
2. HPLC Traces for SynVesT-1 and [^{18}F]SynVest-1 S10–S11
3. ^{1}H and ^{13}C NMR Spectra for all Compounds S12–S35

1. Chiral and Racemic HPLC Traces for compounds **2**, **3**, **SynVesT-1** and **10**

Racemate **2**:

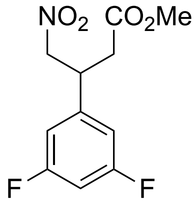

C:\LabSolutions\LCsolution\Sample_Guest\Holly\HM4-045 racemate.lcd

Acquired by : Admin
Sample Name : HM4-045
Sample ID : HM4-045
Injection Volume : 20 uL
Data File Name : HM4-045 racemate.lcd
Method File Name : AD-H_5%iPrOH-Hexane_1mL-min_20min.lcm
Data Acquired : 21/09/2020 14:37:23
Data Processed : 21/09/2020 16:52:58
HM4-045 racemate
AD-H; IPA : Hex = 5 : 95 @ 1mL/min for 20 min

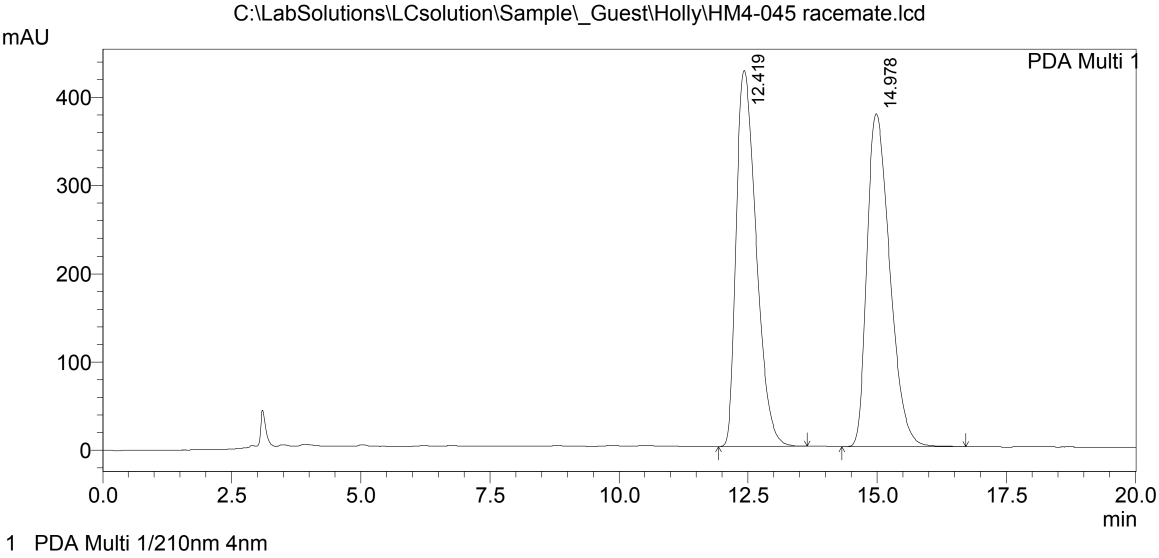

PDA Ch1 210nm 4nm

Peak#	Ret. Time	Area	Height	Area %	Height %
1	12.419	11512761	425916	49.770	53.047
2	14.978	11619386	376989	50.230	46.953
Total		23132147	802905	100.000	100.000

(R)-Enantiomer **2**:

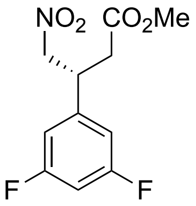

C:\LabSolutions\LCsolution\Sample_Guest\Holly\HM4-083 R-enantiomer.lcd
Acquired by : Admin
Sample Name : HM4-083 R-enantiomer
Sample ID : HM4-083
Injection Volume : 20 uL
Data File Name : HM4-083 R-enantiomer.lcd
Method File Name : AD-H_5%iPrOH-Hexane_1mL-min_20min.lcm
Data Acquired : 21/09/2020 15:00:29
Data Processed : 21/09/2020 17:02:30
HM4-083 R-enantiomer
AD-H; IPA : Hex = 5 : 95 @ 1mL/min for 20 min

C:\LabSolutions\LCsolution\Sample_Guest\Holly\HM4-083 R-enantiomer.lcd

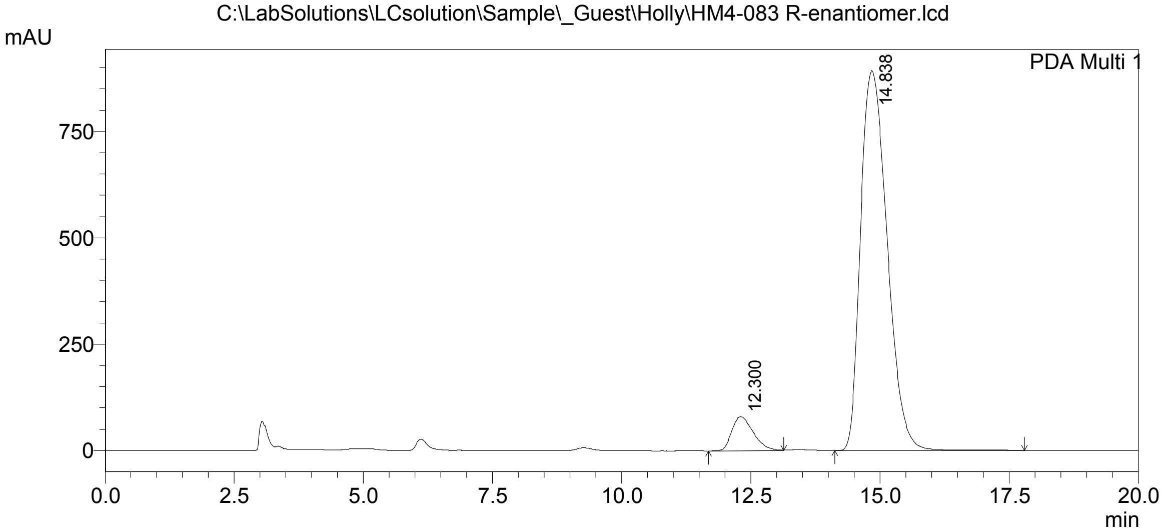

1 PDA Multi 1/210nm 4nm

PDA Ch1 210nm 4nm

Peak#	Ret. Time	Area	Height	Area %	Height %
1	12.300	2344308	80172	6.816	8.236
2	14.838	32050660	893298	93.184	91.764
Total		34394969	973470	100.000	100.000

Racemate **3**:

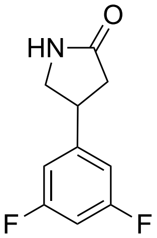

C:\LabSolutions\LCsolution\Sample_Guest\Holly\HM4-051 racemate.lcd

Acquired by	: Admin
Sample Name	: HM4-051 racemate
Sample ID	: HM4-051 racemate
Injection Volume	: 20 uL
Data File Name	: HM4-051 racemate.lcd
Method File Name	: AD-H_5%iPrOH-Hexane_1mL-min_30min.lcm
Data Acquired	: 21/09/2020 15:26:07
Data Processed	: 21/09/2020 17:05:10

HM4-051 racemate
AD-H; IPA : Hex = 5 : 95 @ 1mL/min for 30 min

C:\LabSolutions\LCsolution\Sample_Guest\Holly\HM4-051 racemate.lcd

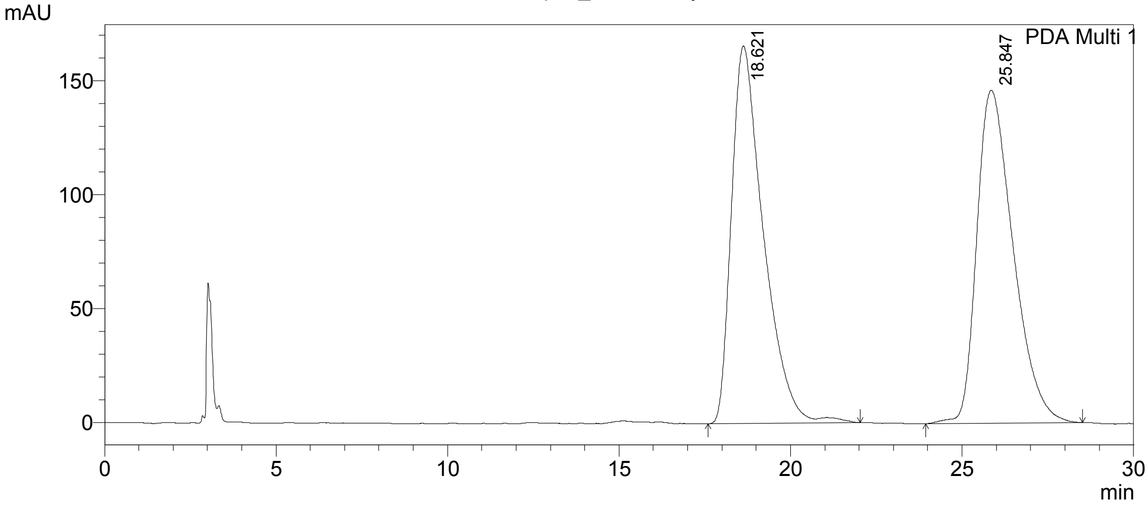

1 PDA Multi 1/210nm 4nm

PDA Ch1 210nm 4nm

Peak#	Ret. Time	Area	Height	Area %	Height %
1	18.621	10707026	165585	49.838	53.127
2	25.847	10776588	146094	50.162	46.873
Total		21483614	311679	100.000	100.000

(*R*)-Enantiomer **3**:

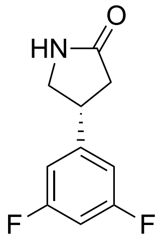

C:\LabSolutions\LCsolution\Sample_Guest\Holly\HM4-084 R-enantiomer.lcd

Acquired by : Admin
Sample Name : HM4-084 R-enantiomer
Sample ID : HM4-084 R-enantiomer
Injection Volume : 20 uL
Data File Name : HM4-084 R-enantiomer.lcd
Method File Name : AD-H_5%iPrOH-Hexane_1mL-min_30min.lcm
Data Acquired : 21/09/2020 16:04:56
Data Processed : 21/09/2020 17:09:50
HM4-084 R-enantiomer
AD-H; IPA : Hex = 5 : 95 @ 1mL/min for 30 min

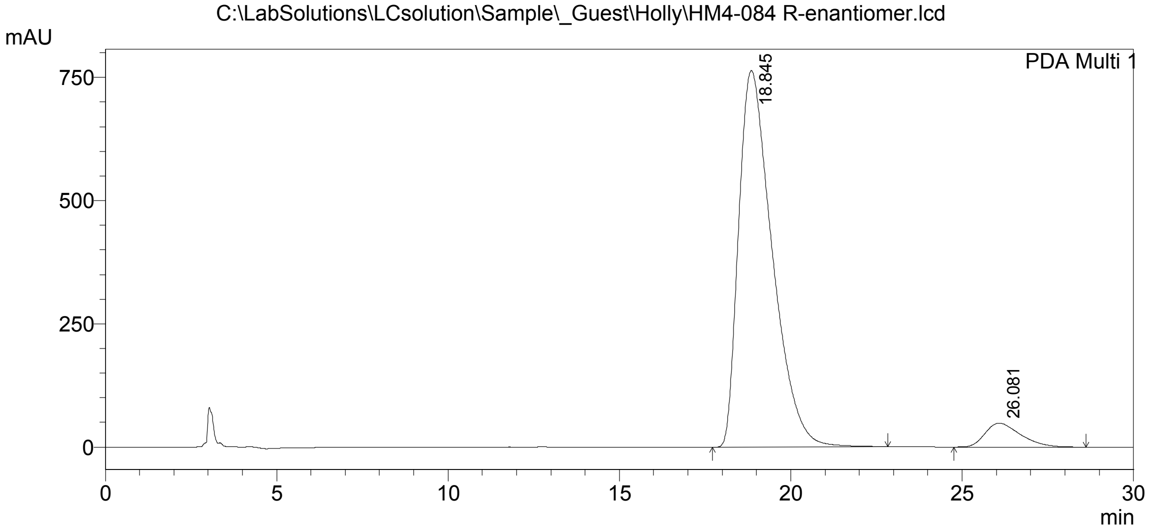

1 PDA Multi 1/210nm 4nm

PDA Ch1 210nm 4nm

Peak#	Ret. Time	Area	Height	Area %	Height %
1	18.845	52112355	764235	93.708	94.021
2	26.081	3498905	48603	6.292	5.979
Total		55611260	812838	100.000	100.000

Racemate of **SynVesT-1**:

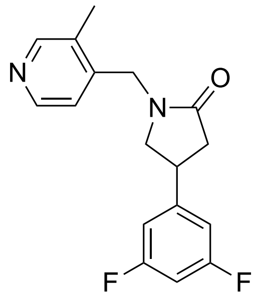

C:\LabSolutions\LCsolution\Sample_Clark_Guest\Holly\HM4-057 inj4.lcd

Acquired by	: Admin
Sample Name	: HM4-057 inj4
Sample ID	: HM4-057 inj4
Injection Volume	: 20 uL
Data File Name	: HM4-057 inj4.lcd
Method File Name	: AD-H_10%iPrOH-Hexane_2mL-min_40min.lcm
Data Acquired	: 21/09/2022 14:01:00
Data Processed	: 21/09/2022 14:52:05

AD-H column
2 mL/min
10% IPA / 90% Hexane

C:\LabSolutions\LCsolution\Sample_Clark_Guest\Holly\HM4-057 inj4.lcd

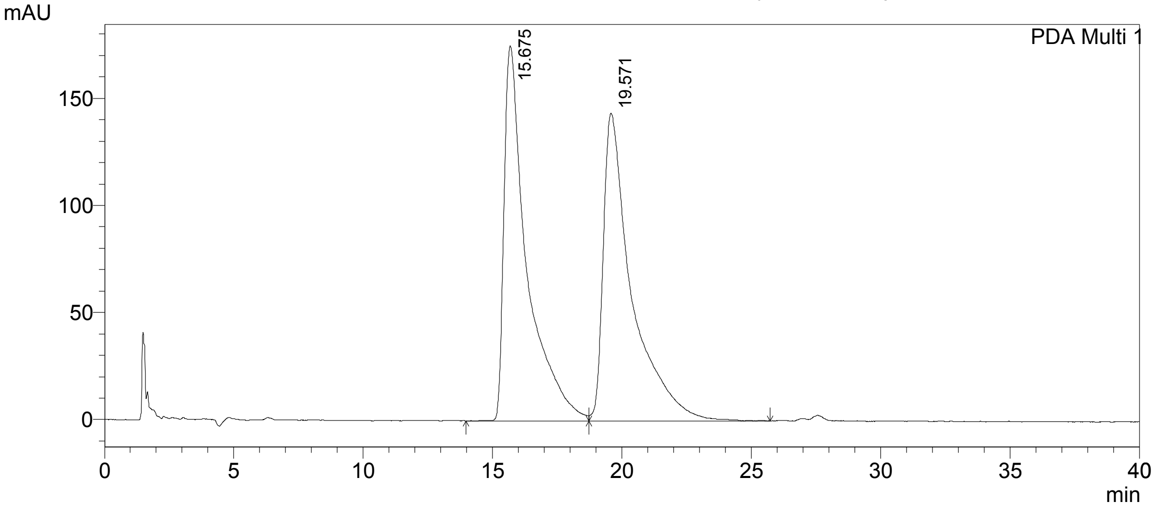

1 PDA Multi 1/210nm 4nm

PDA Ch1 210nm 4nm

Peak#	Ret. Time	Area	Height	Area %	Height %
1	15.675	10782036	175249	49.504	54.945
2	19.571	10997982	143703	50.496	45.055
Total		21780019	318952	100.000	100.000

(*R*)-Enantiomer of **SynVesT-1**:

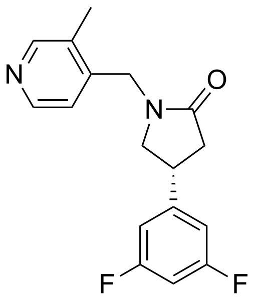

C:\LabSolutions\LCsolution\Sample_Clark_Guest\Holly\VB2-26 inj1.lcd

Acquired by : Admin
Sample Name : VB2-26 inj1
Sample ID : VB2-26 inj1
Injection Volume : 20 uL
Data File Name : VB2-26 inj1.lcd
Method File Name : AD-H_10%iPrOH-Hexane_2mL-min_40min.lcm
Data Acquired : 21/09/2022 14:46:22
Data Processed : 21/09/2022 15:43:36
AD-H column
2 mL/min
10% IPA / 90% Hexane

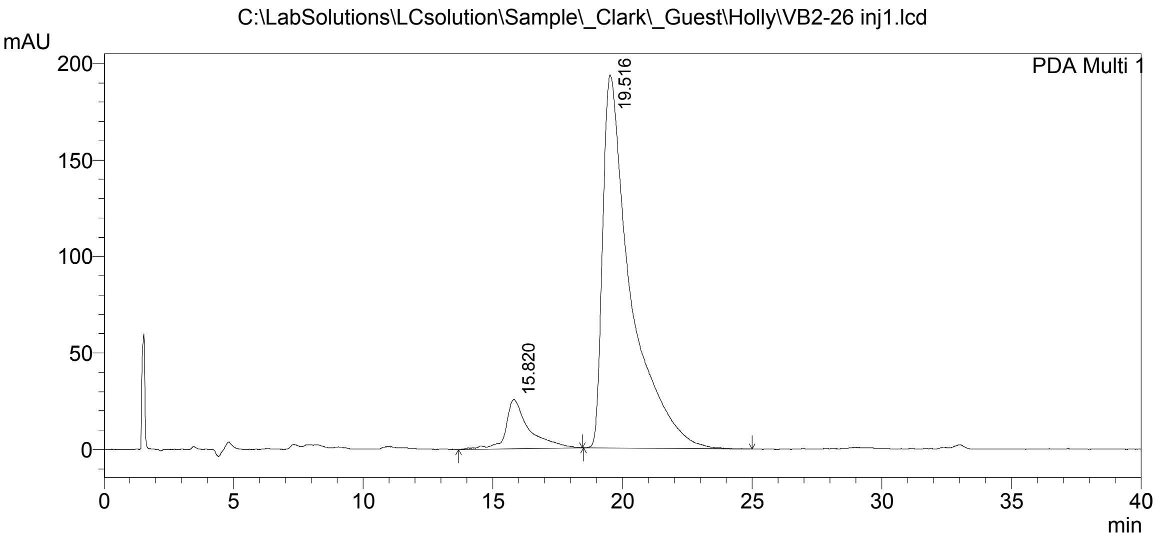

1 PDA Multi 1/210nm 4nm

PDA Ch1 210nm 4nm

Peak#	Ret. Time	Area	Height	Area %	Height %
1	15.820	1578264	25385	9.855	11.622
2	19.516	14436532	193029	90.145	88.378
Total		16014795	218414	100.000	100.000

Racemate **10**:

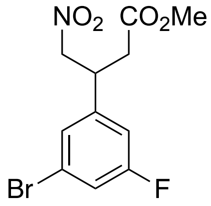

C:\LabSolutions\LCsolution\Sample_Guest\Holly\HM4-056 racemate inj6.lcd
Acquired by : Admin
Sample Name : HM4-056 racemate inj6
Sample ID : HM4-056 racemate inj6
Injection Volume : 20 uL
Data File Name : HM4-056 racemate inj6.lcd
Method File Name : AD-H_2%iPrOH-Hexane_1mL-min_30min.lcm
Data Acquired : 23/09/2020 16:08:48
Data Processed : 23/09/2020 16:38:50
HM4-056 racemate inj6
AD-H; IPA : Hex = 2 : 98 @ 1mL/min 30 min

C:\LabSolutions\LCsolution\Sample_Guest\Holly\HM4-056 racemate inj6.lcd

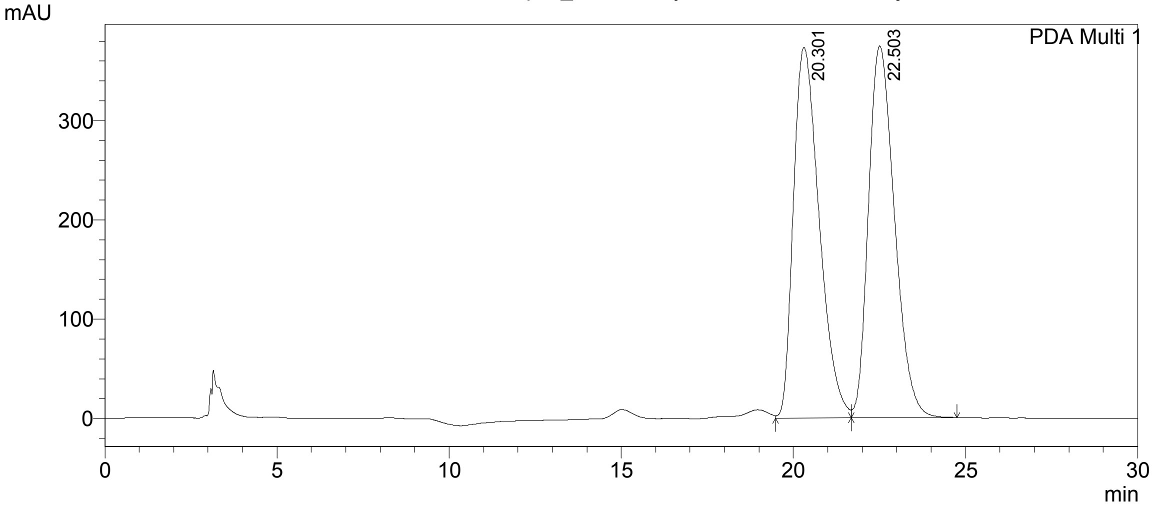

1 PDA Multi 1/210nm 4nm

PDA Ch1 210nm 4nm

Peak#	Ret. Time	Area	Height	Area %	Height %
1	20.301	19520939	373575	49.771	49.925
2	22.503	19700943	374695	50.229	50.075
Total		39221882	748270	100.000	100.000

(*R*)-Enantiomer **10**:

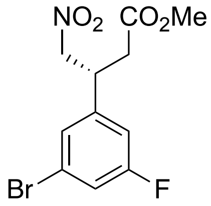

C:\LabSolutions\LCsolution\Sample_Guest\Holly\HM4-093 R-enantiomer inj2.lcd

Acquired by	: Admin
Sample Name	: HM4-093 R-enantiomer inj2
Sample ID	: HM4-093 R-enantiomer inj2
Injection Volume	: 20 uL
Data File Name	: HM4-093 R-enantiomer inj2.lcd
Method File Name	: AD-H_2%iPrOH-Hexane_1mL-min_30min.lcm
Data Acquired	: 23/09/2020 14:56:35
Data Processed	: 23/09/2020 17:18:16

HM4-093 R-enantiomer inj2
AD-H; IPA : Hex = 2 : 98 @ 1mL/min 30 min

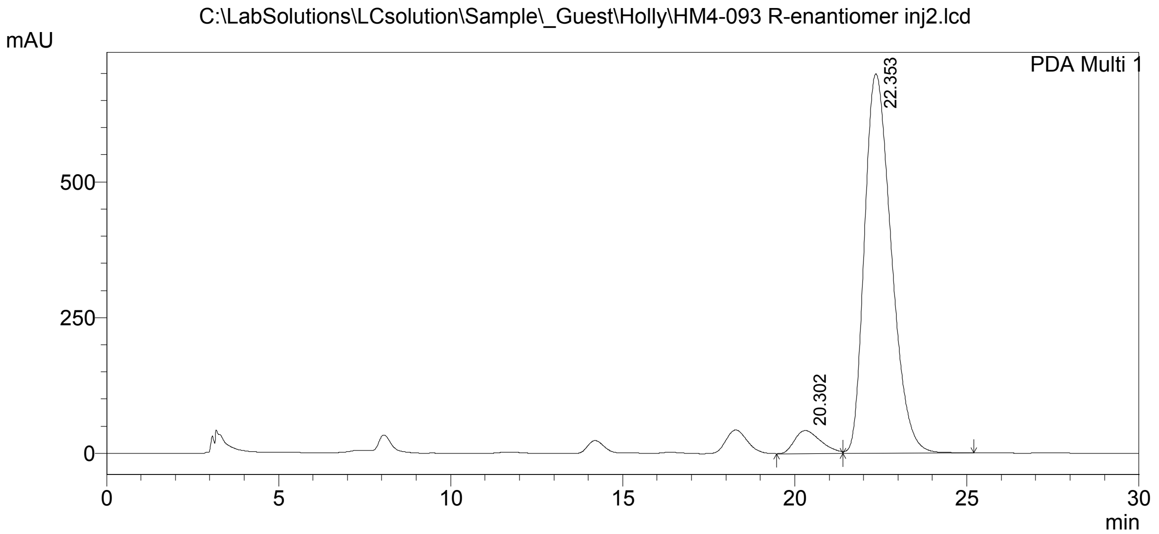

1 PDA Multi 1/210nm 4nm

PDA Ch1 210nm 4nm

Peak#	Ret. Time	Area	Height	Area %	Height %
1	20.302	2249598	42341	5.608	5.712
2	22.353	37867036	698940	94.392	94.288
Total		40116634	741281	100.000	100.000

2. HPLC Traces for SynVesT-1 and [^{18}F]SynVest-1

SynVesT-1 (UV standard):

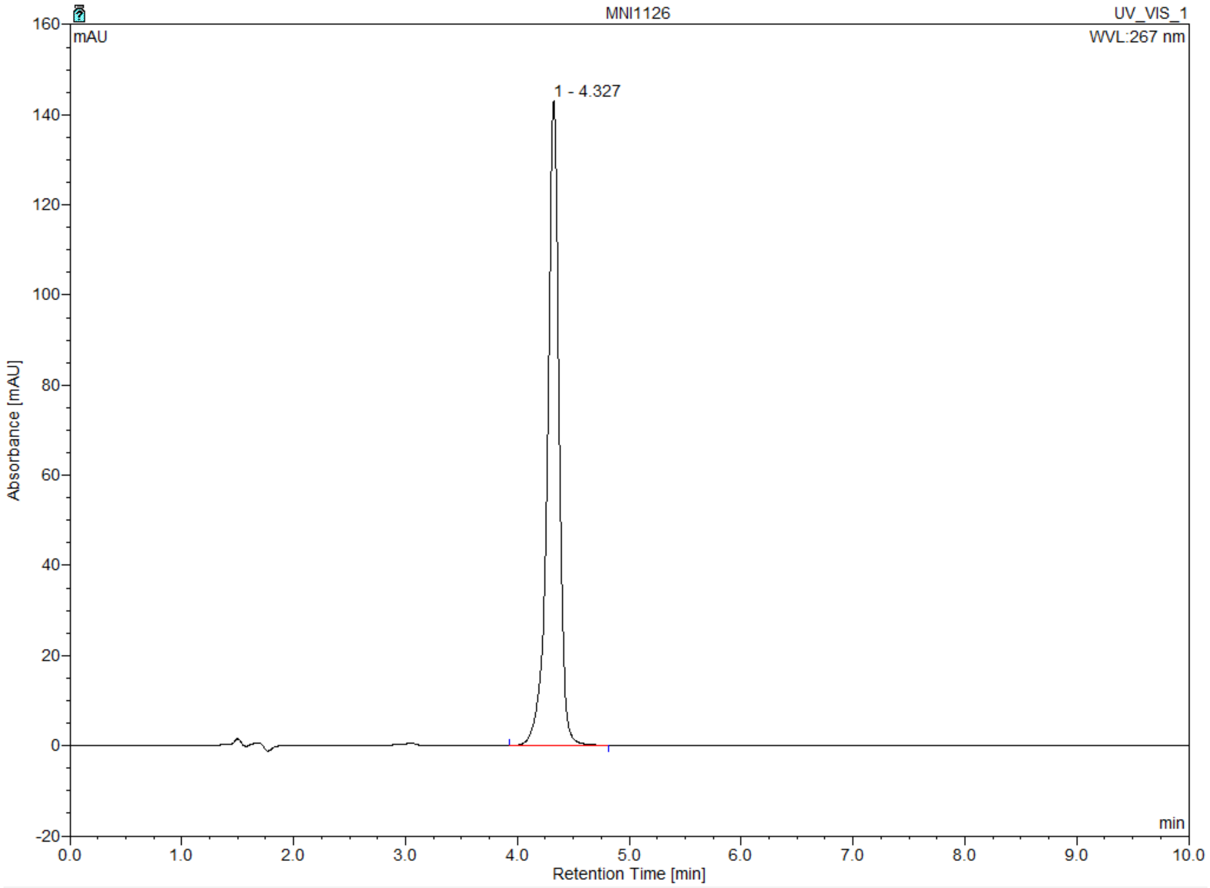

A	B	C	D	E	F	G	H	I	J	K	L	M	N
No.	Peakname	Ret.Time min	Area mAU*min	Amount	Type	Height mAU	Rel.Area %	Resolution					
1	n.a.	4.327	17.2375	n.a.	BMB*	142.927	100.00	n.a.					
Total:			17.2375	0.0000		142.927	100.00						

[^{18}F]SynVesT-1 (Blue: UV; Black: Gamma)

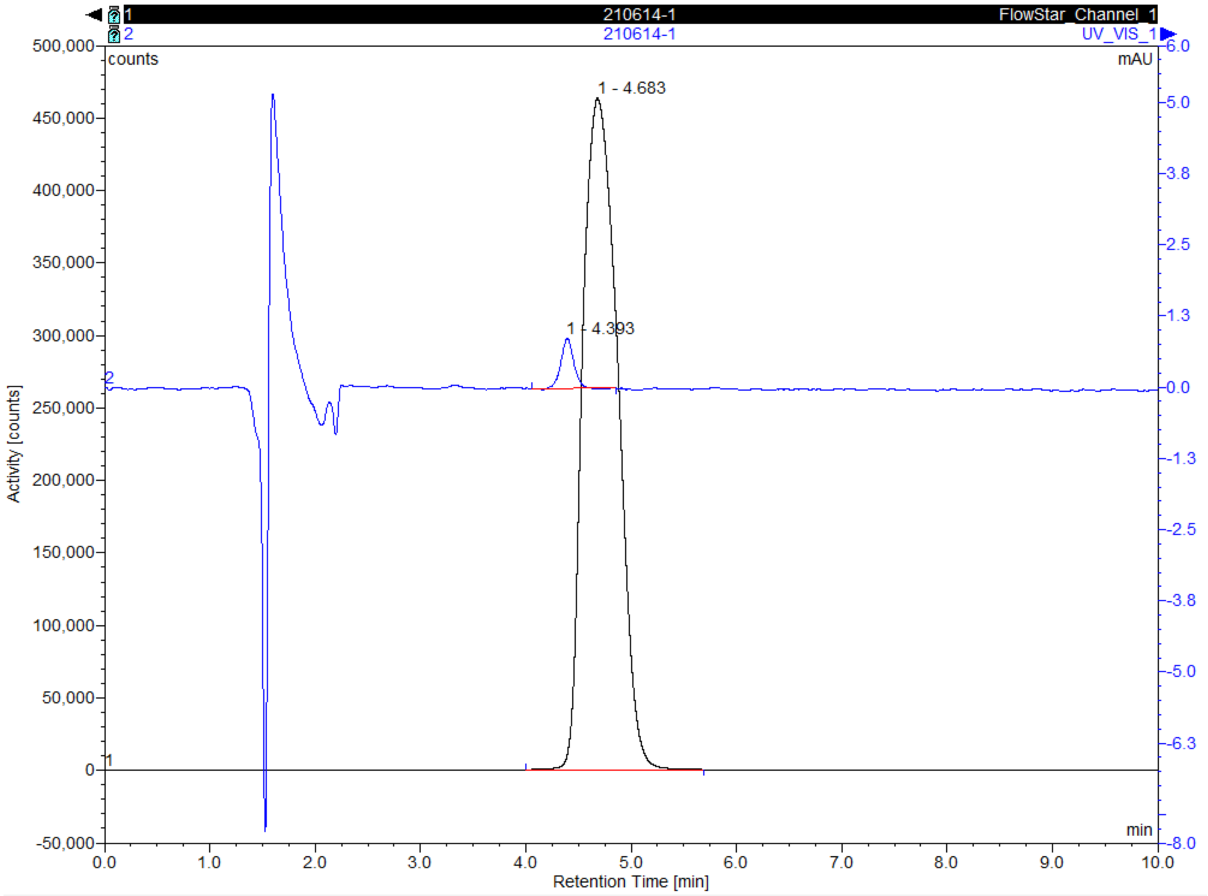

A	B	C	D	E	F	G	H	I	J	K	L	M	N
No.	Peakname	Ret.Time min	Area *min	Amount	Type	Height	Rel.Area %	Resolution					
1	n.a.	4.683	#########	n.a.	BMB*	##########	100.00	n.a.					
Total:			#########	0.0000		##########	100.00						

1. ^{1}H and ^{13}C NMR Spectra for all Compounds

^{1}H NMR (500 MHz, $CDCl_3$)

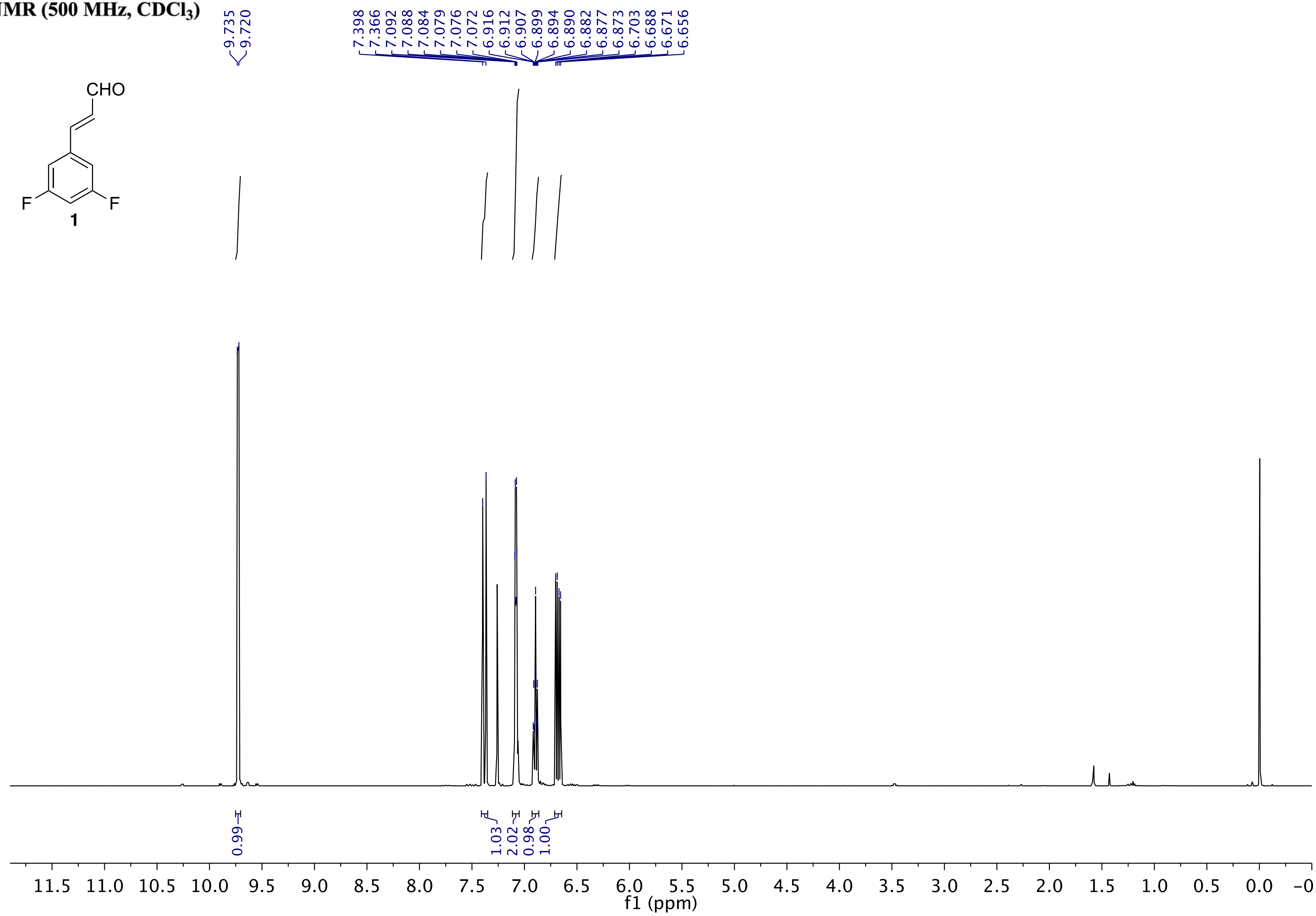

$^{13}C\{^1H\}$ NMR (126 MHz, $CDCl_3$)

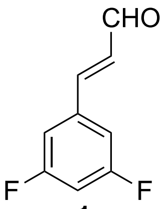

1

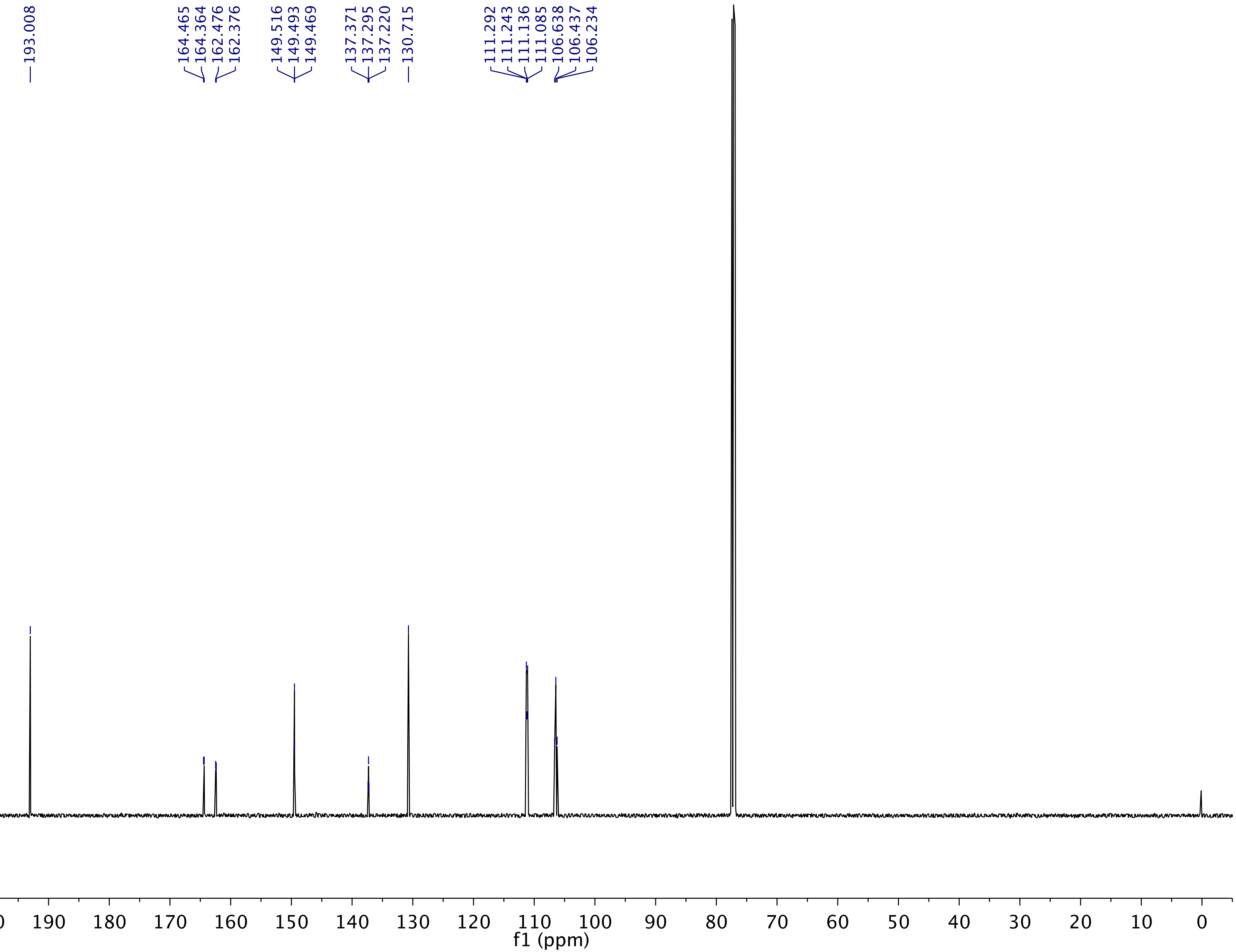

^{1}H NMR (400 MHz, $CDCl_3$)

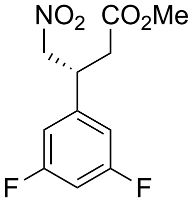

2

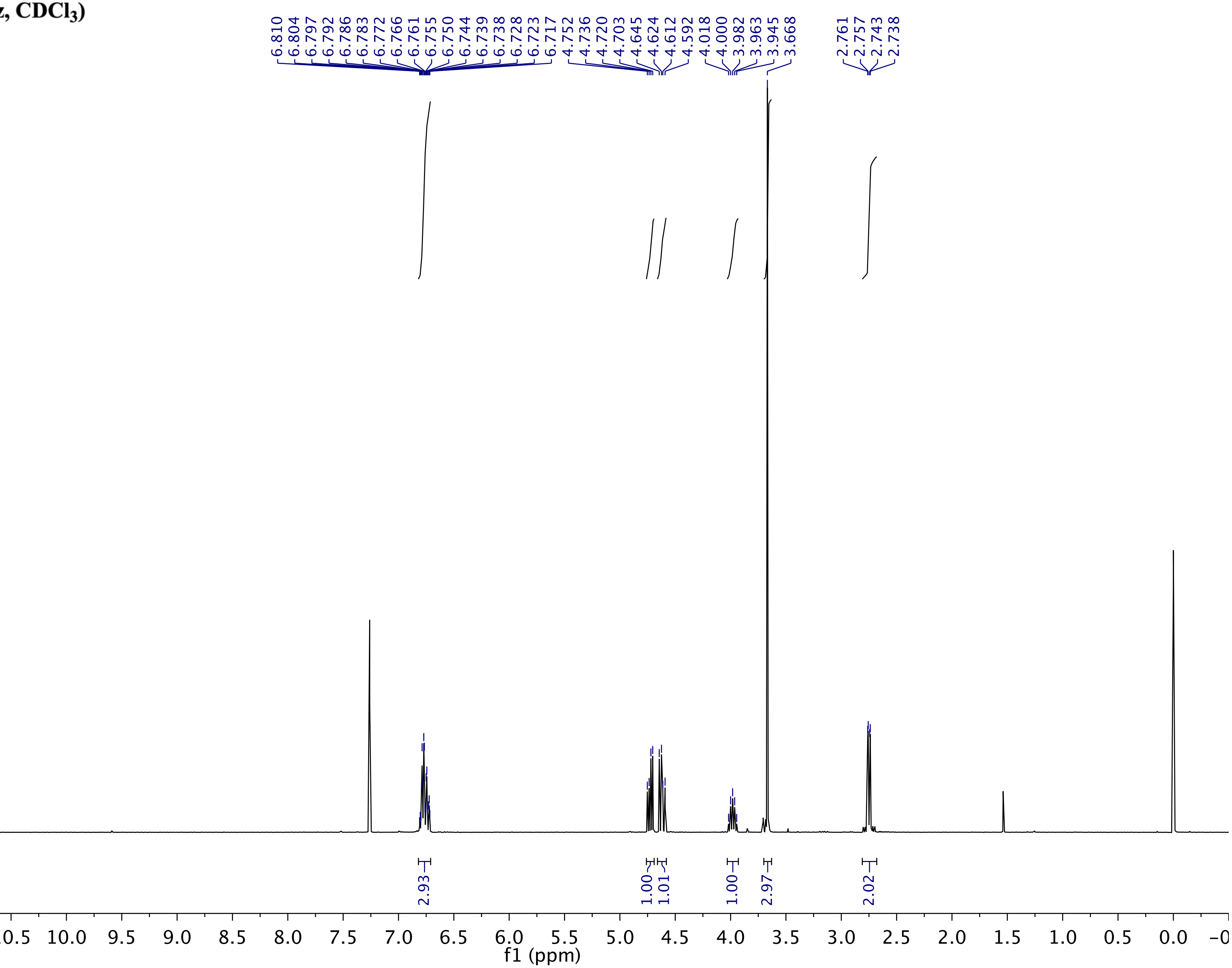

$^{13}C\{^{1}H\}$ **NMR (101 MHz, $CDCl_3$)**

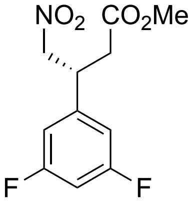

2

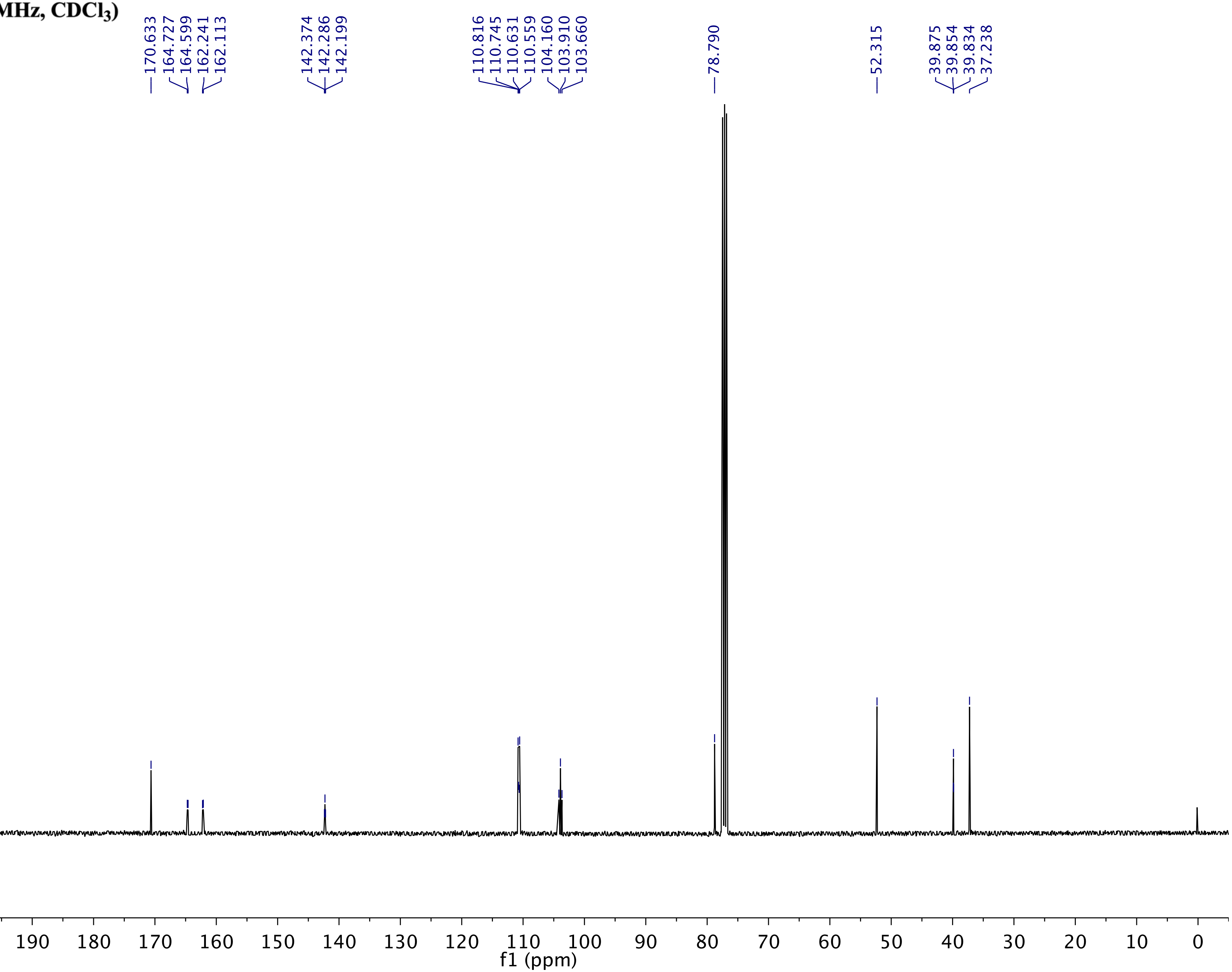

^{1}H NMR (400 MHz, $CDCl_3$)

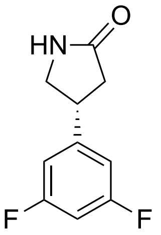

3

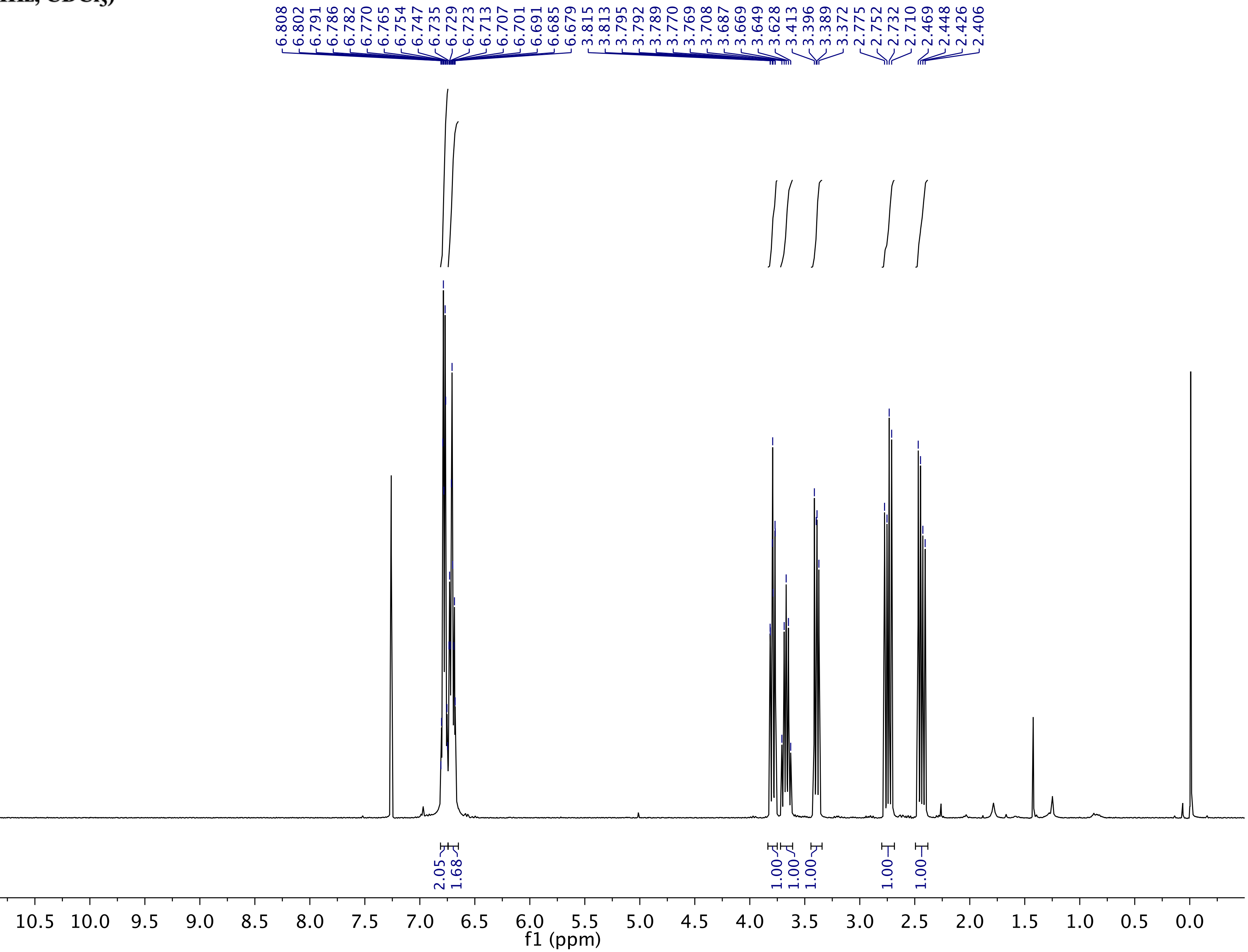

$^{13}C\{^1H\}$ NMR (101 MHz, $CDCl_3$)

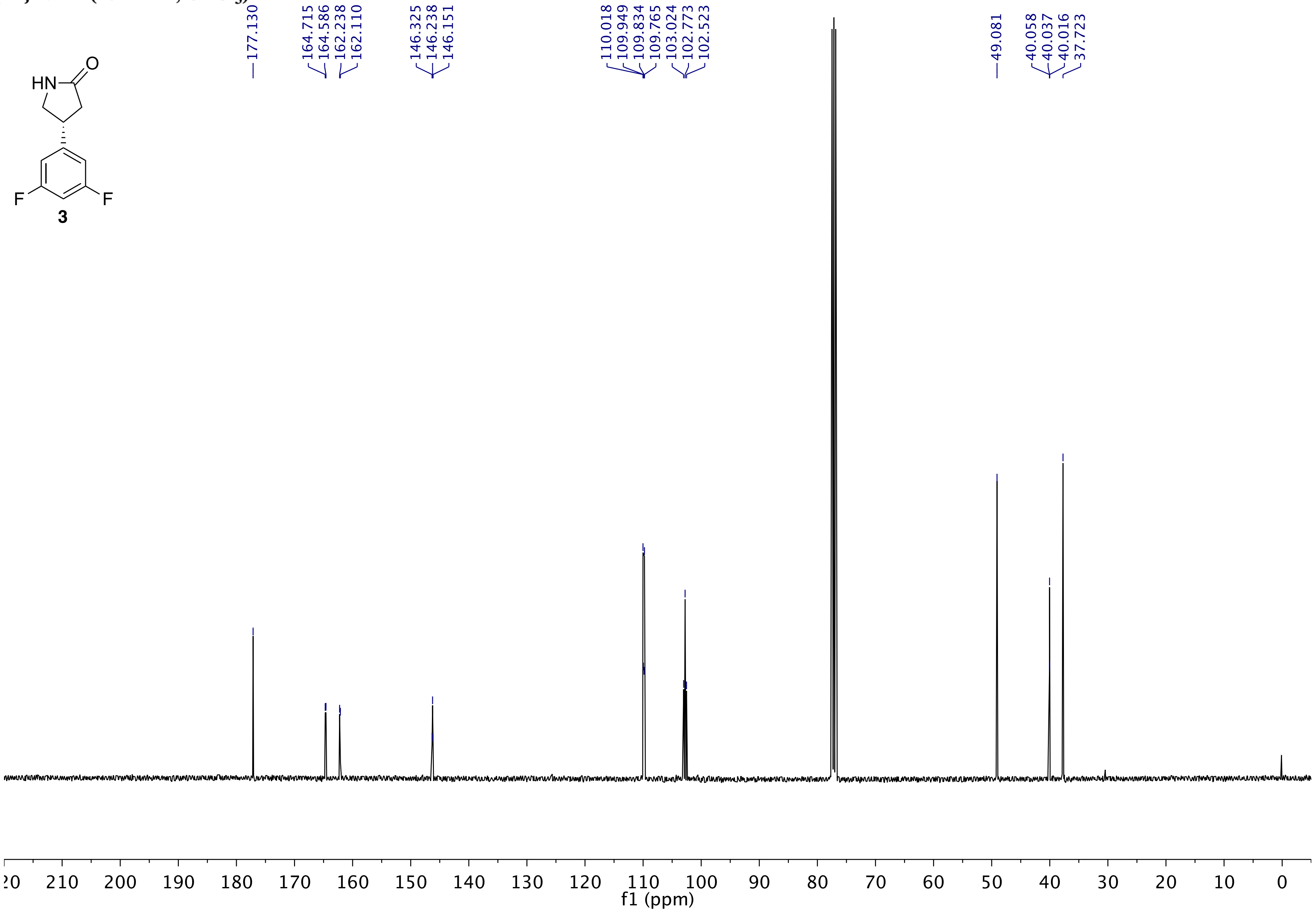

^{1}H NMR (400 MHz, $CDCl_3$)

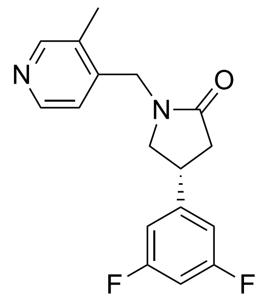

SynVesT-1

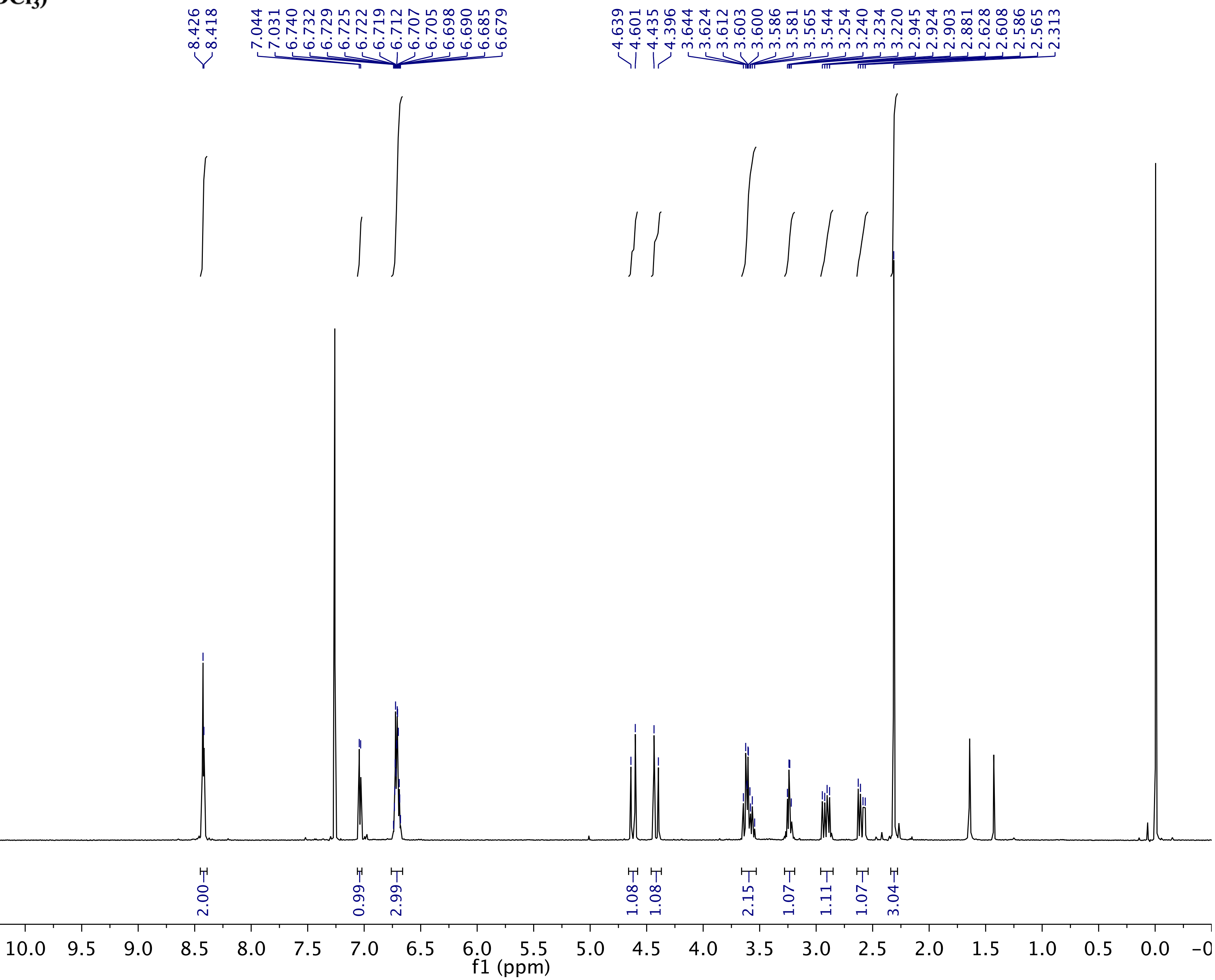

$^{13}C\{^{1}H\}$ NMR (101 MHz, $CDCl_3$)

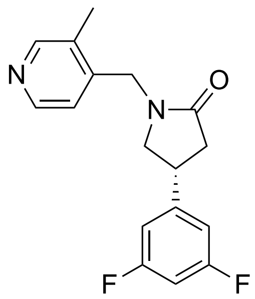

SynVesT-1

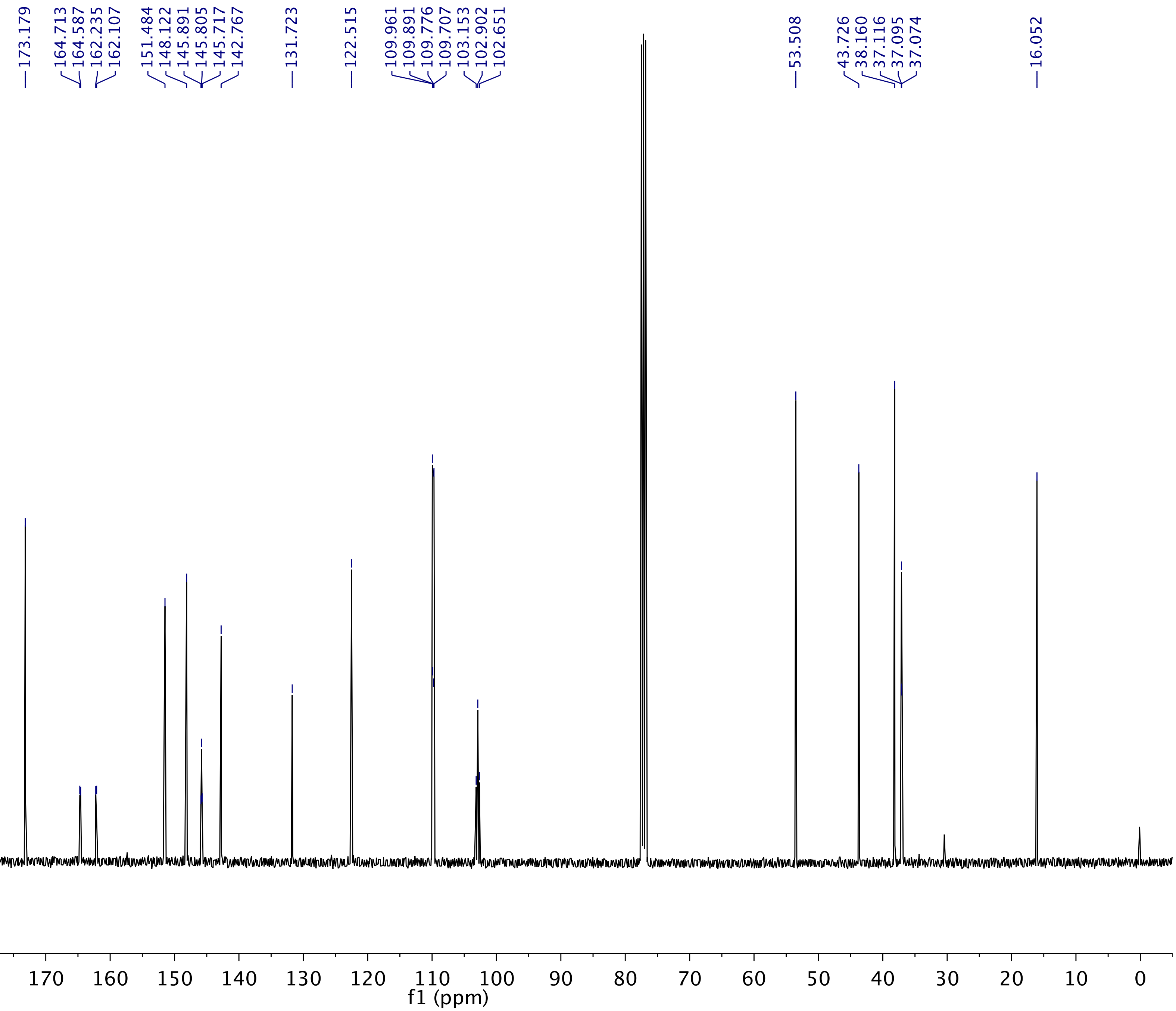

^{1}H NMR (500 MHz, $CDCl_3$)

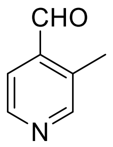

5

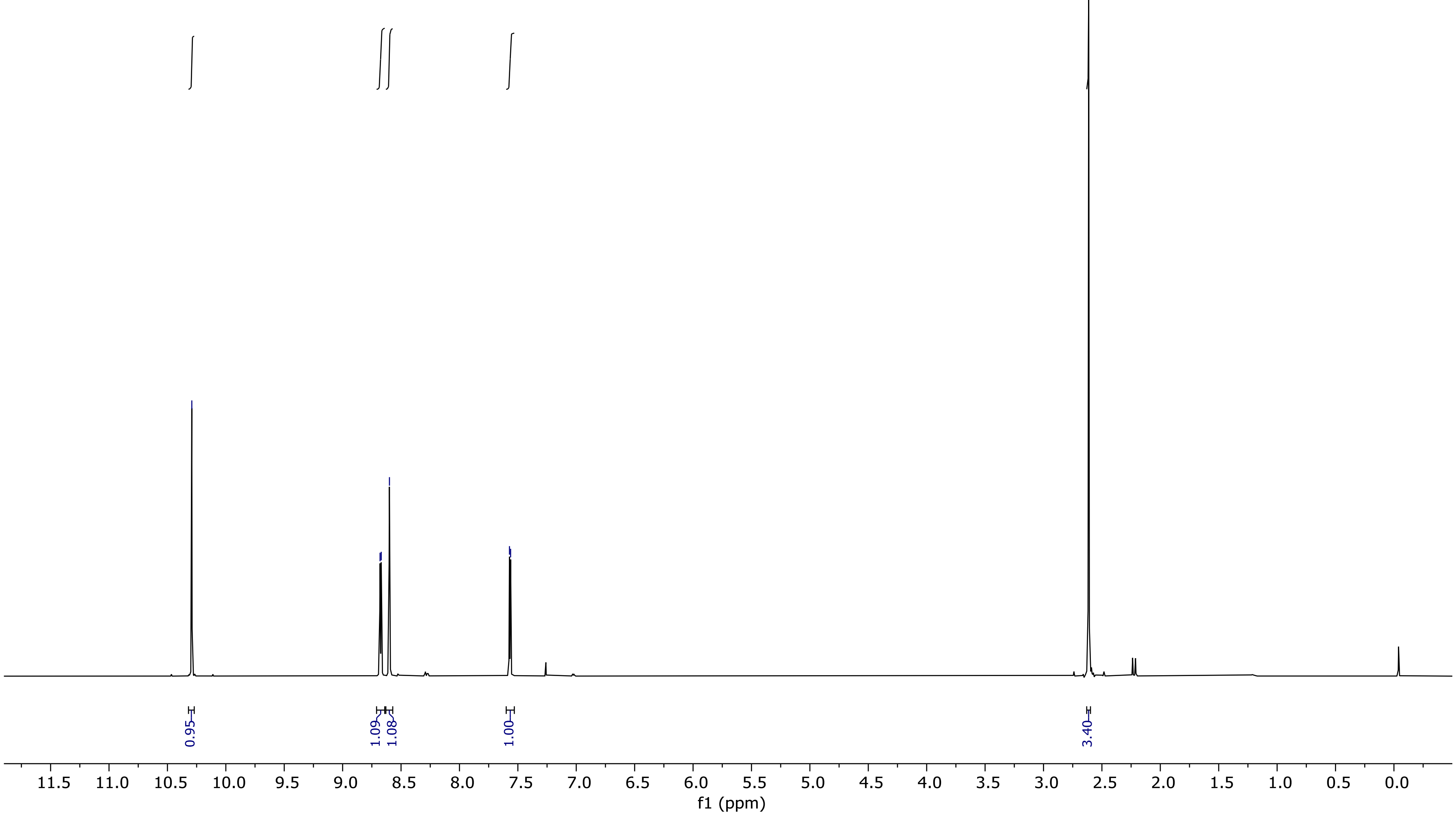

$^{13}C\{^1H\}$ NMR (126 MHz, $CDCl_3$)

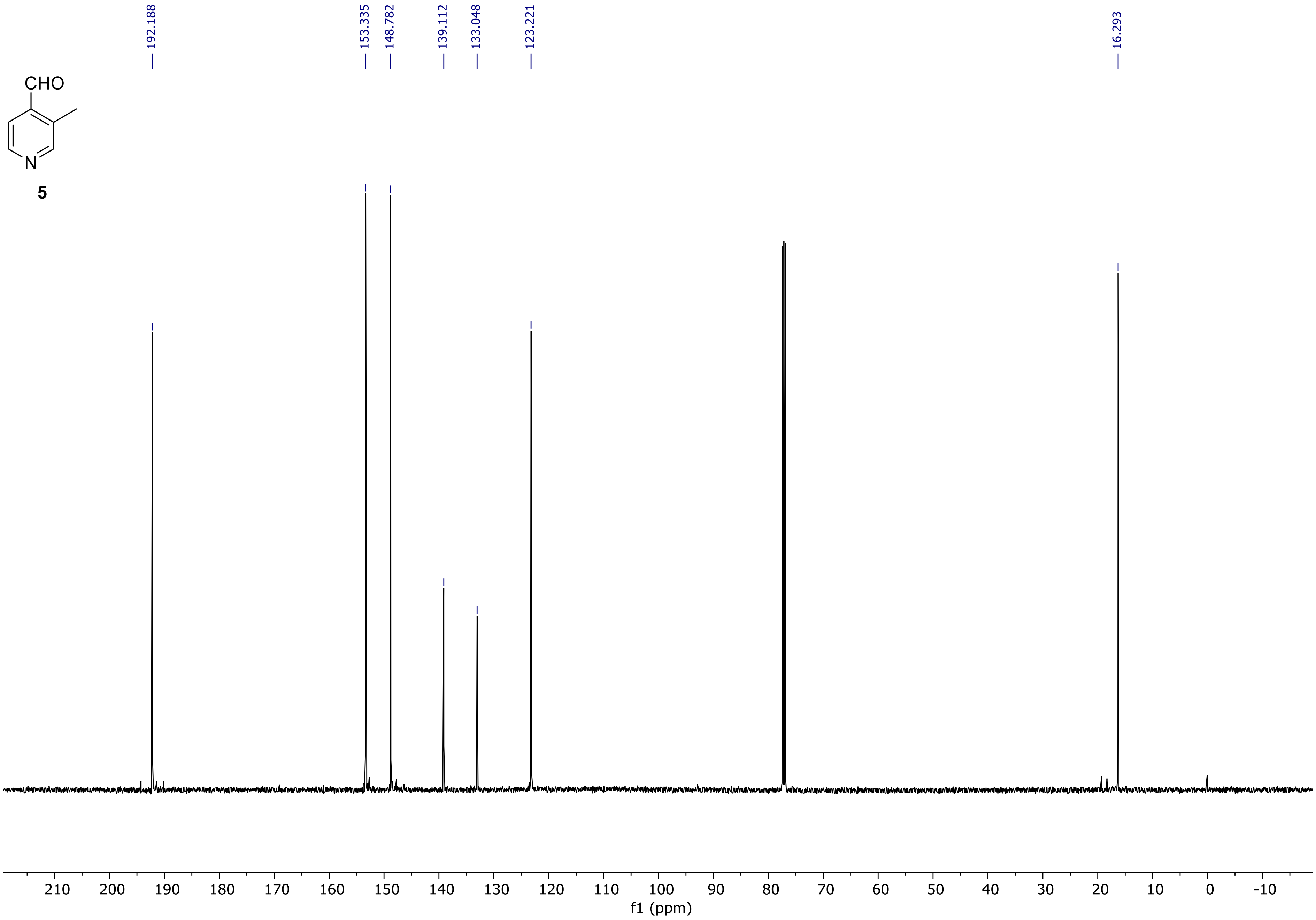

^{1}H NMR (500 MHz, DMSO-d_6)

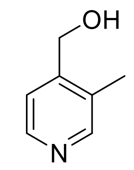

6

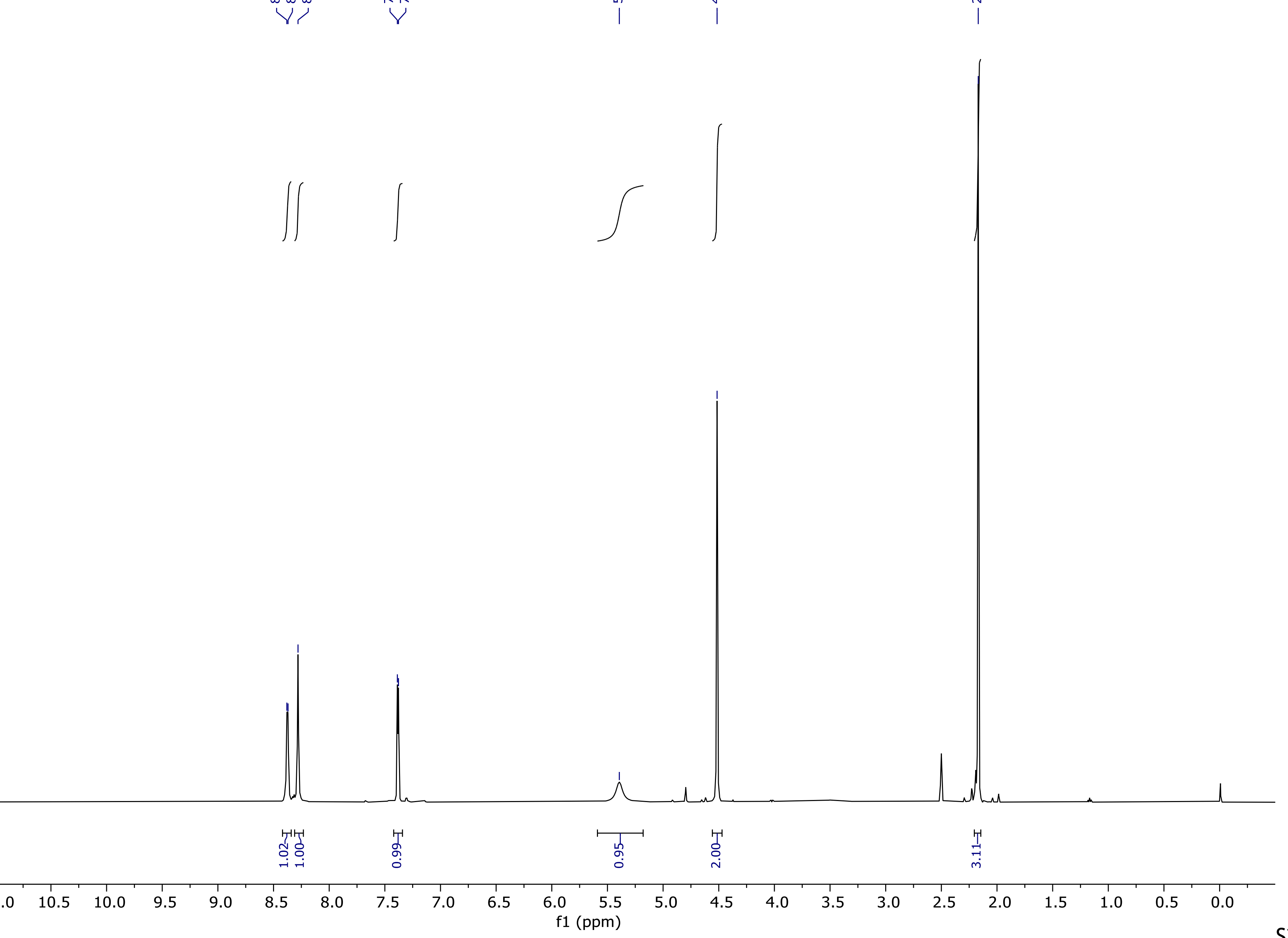

$^{13}C\{^{1}H\}$ NMR (126 MHz, DMSO-d_6)

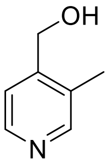

6

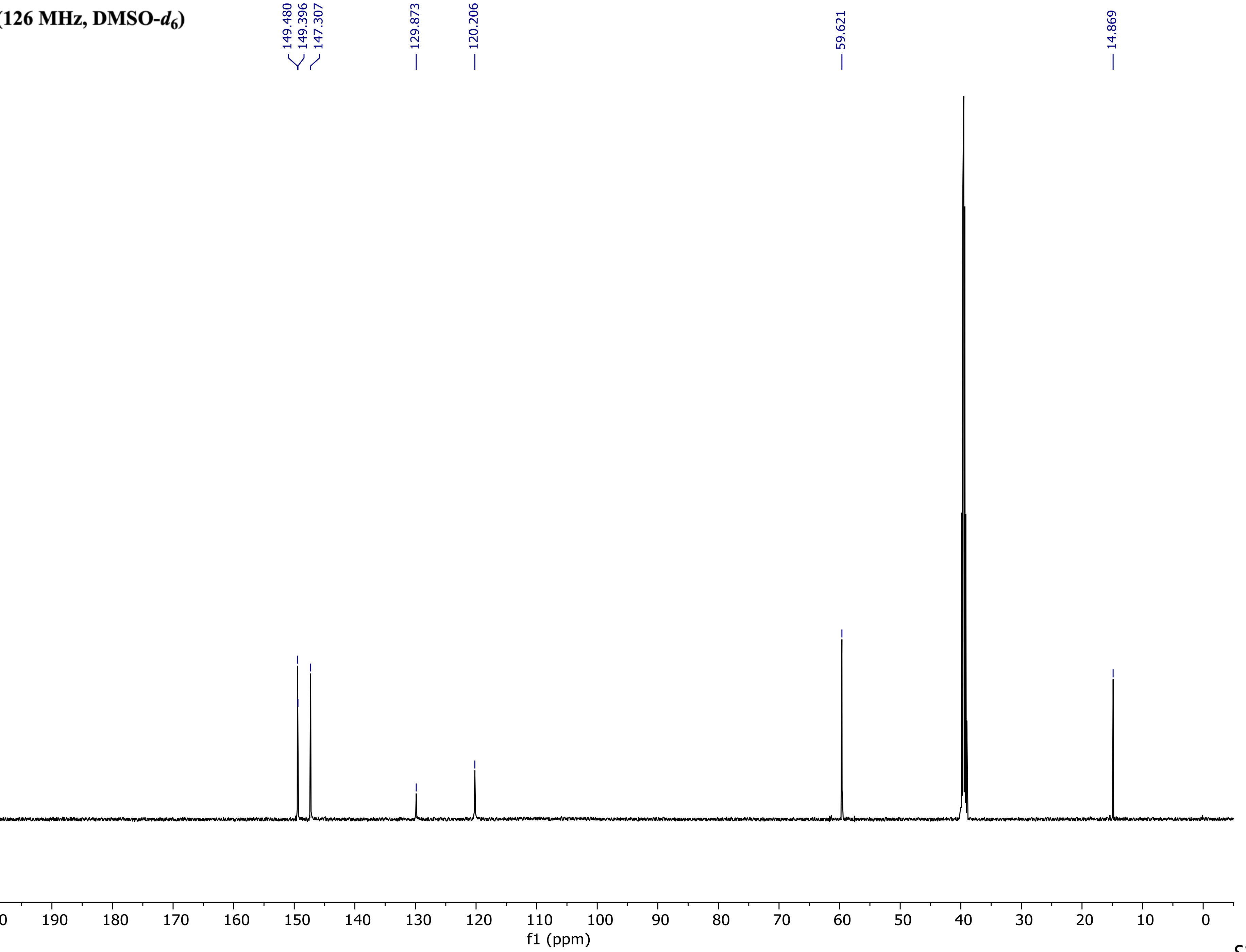

^{1}H NMR (400 MHz, DMSO-d_6)

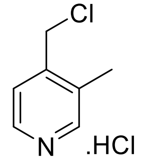

7

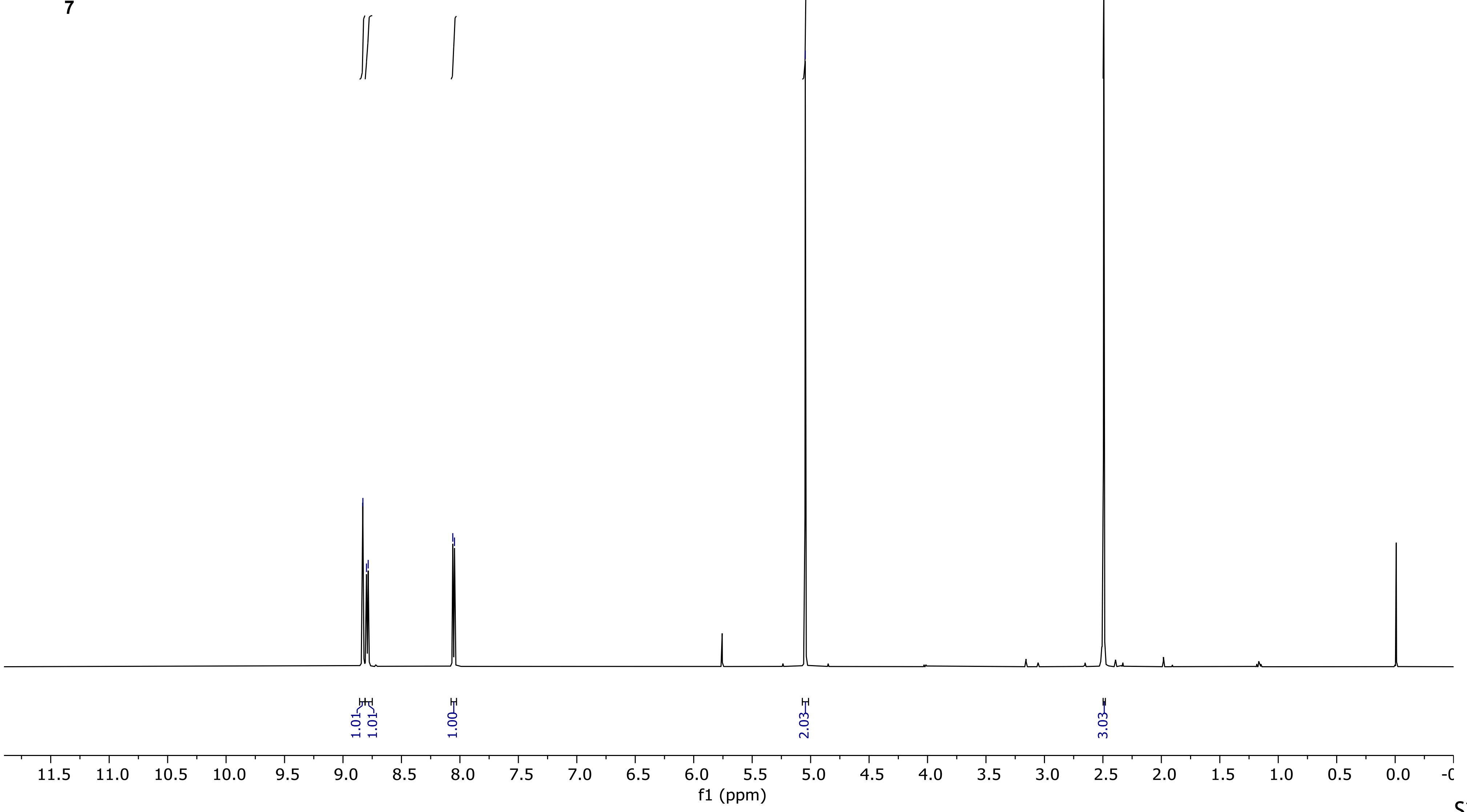

$^{13}C\{^1H\}$ NMR (101 MHz, DMSO-d_6)

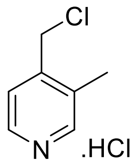

7

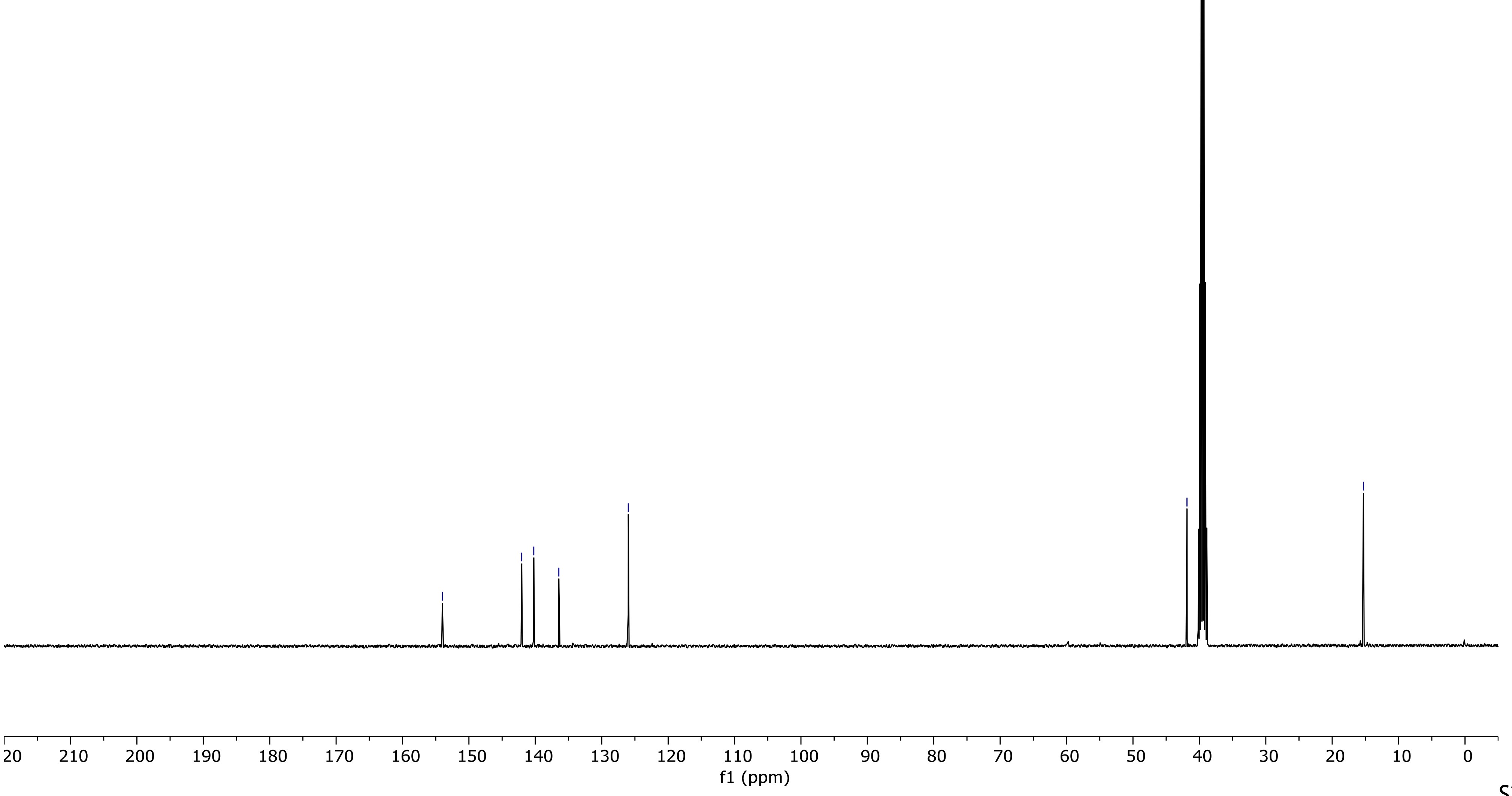

^{1}H NMR (400 MHz, CDCl$_3$)

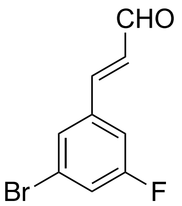

9

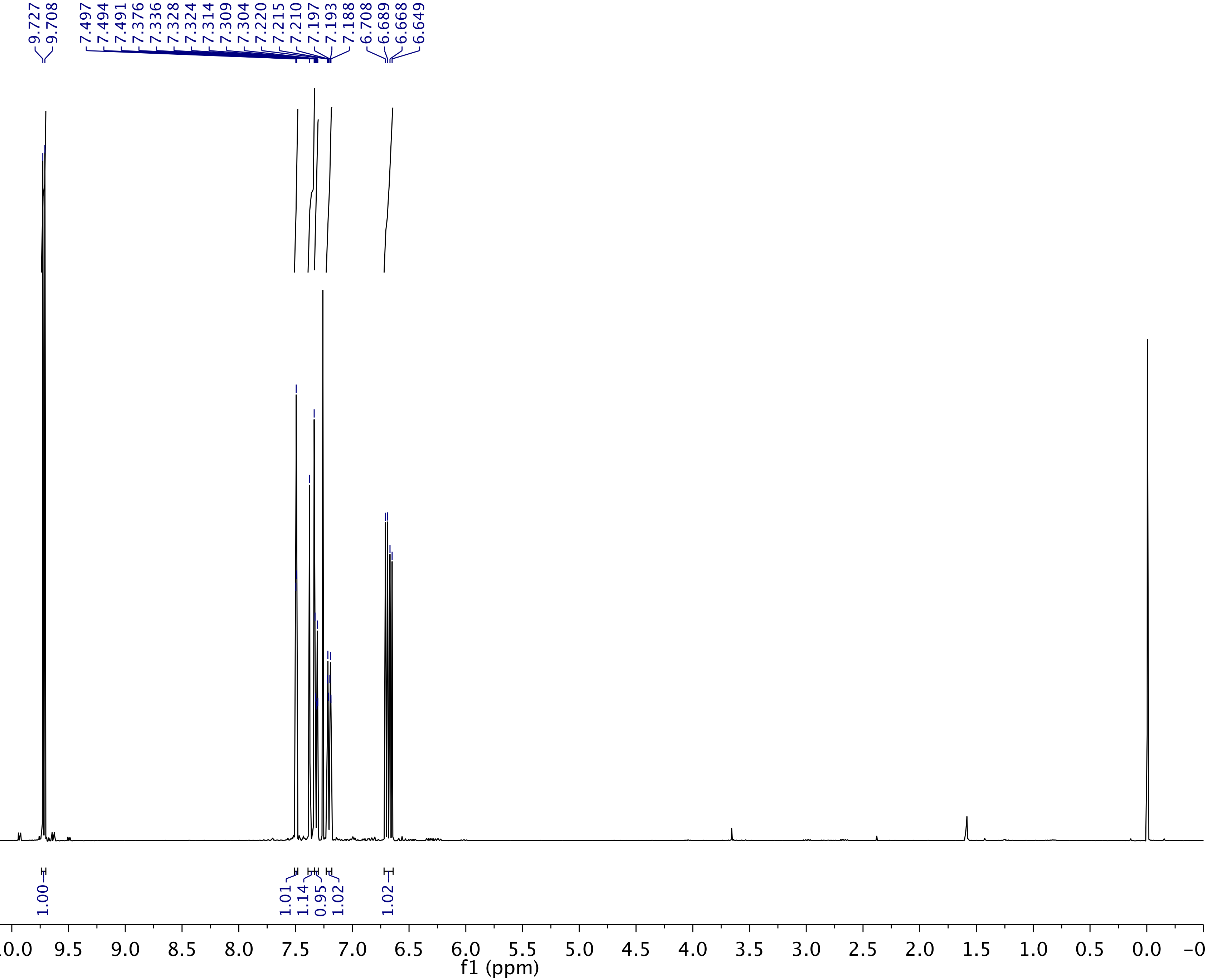

$^{13}C\{^{1}H\}$ NMR (101 MHz, $CDCl_3$)

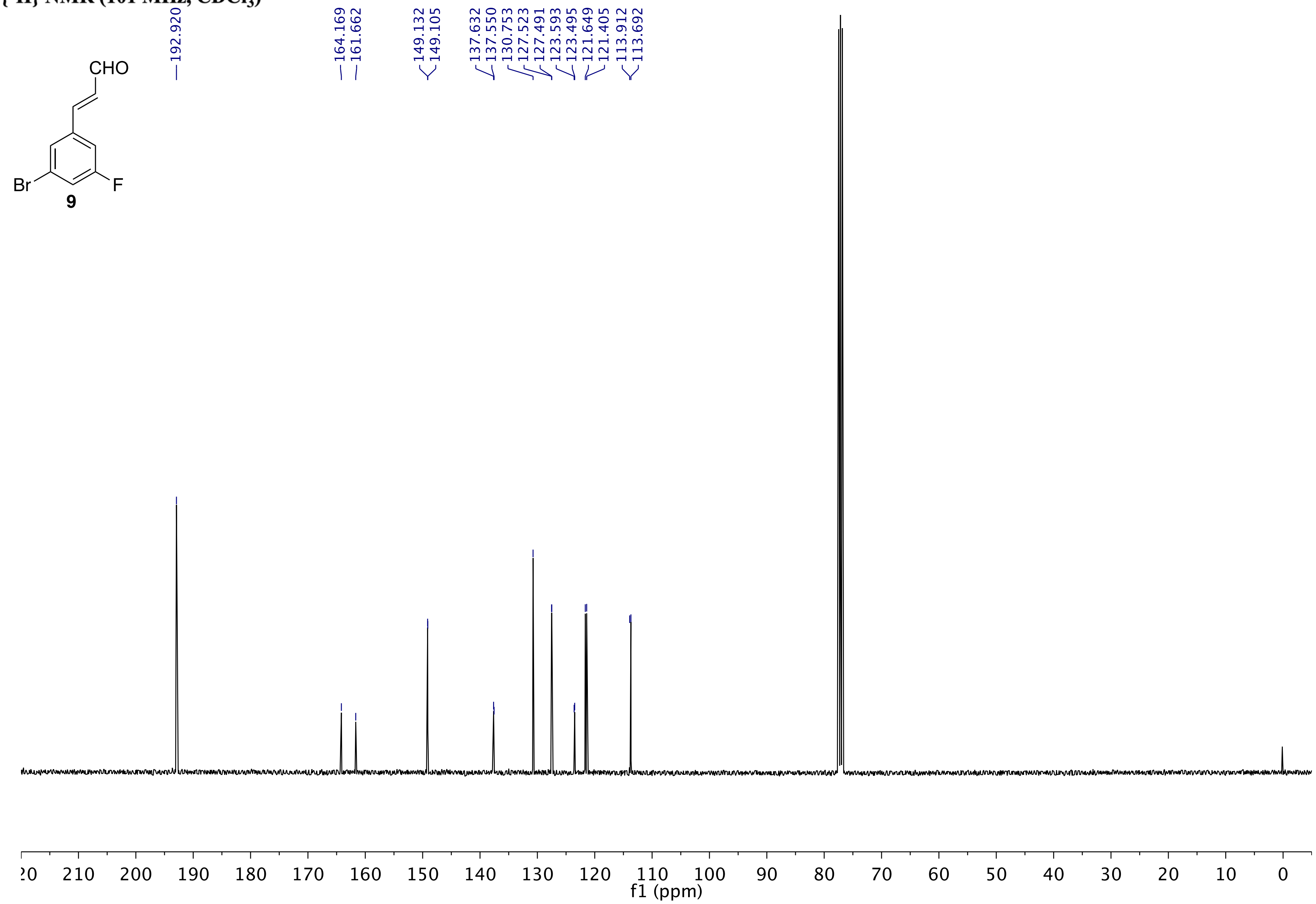

^{1}H NMR (400 MHz, CDCl$_3$)

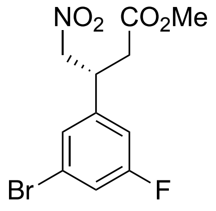

10

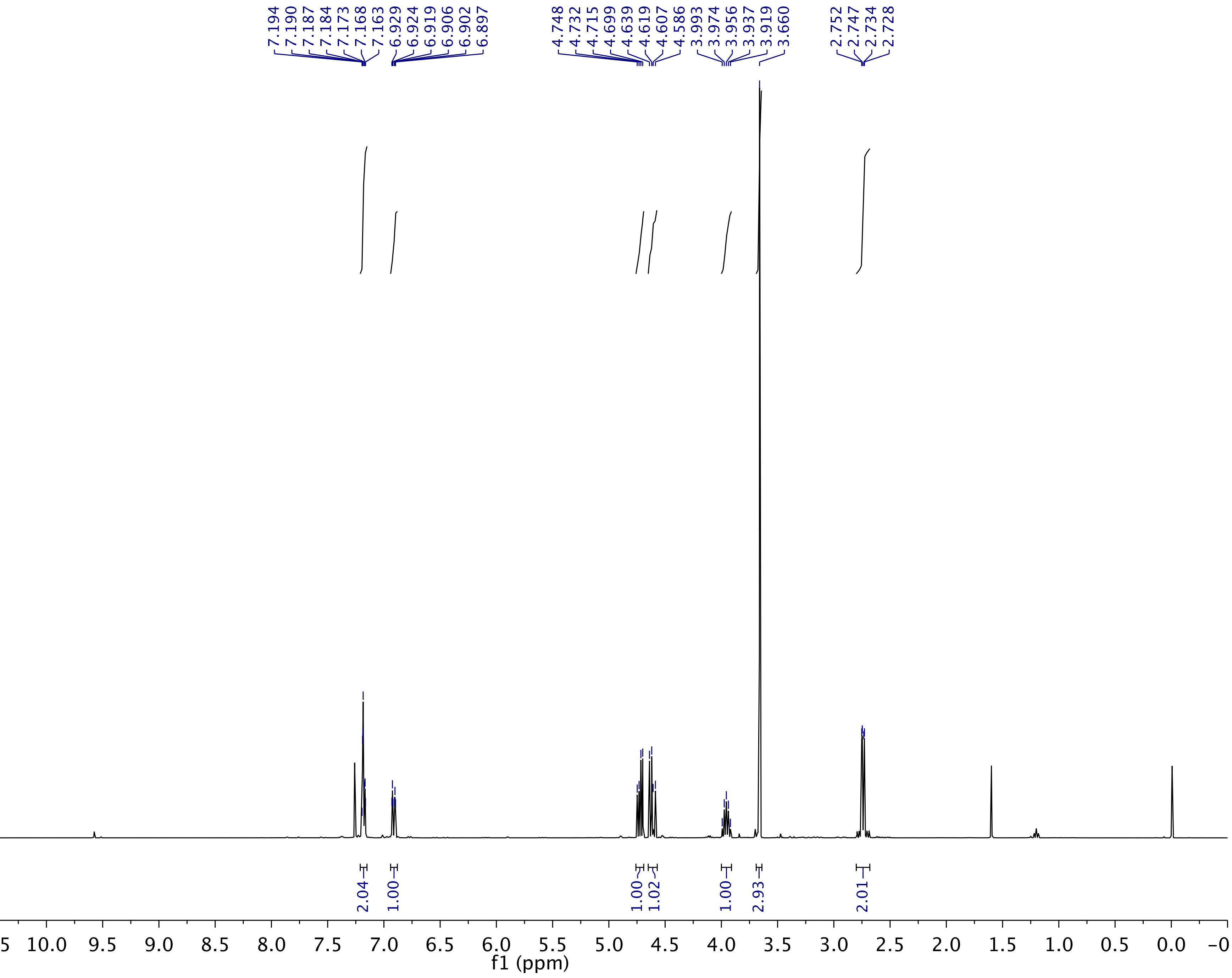

$^{13}C\{^1H\}$ NMR (101 MHz, $CDCl_3$)

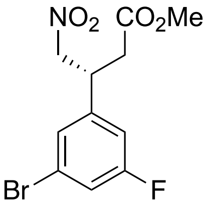

10

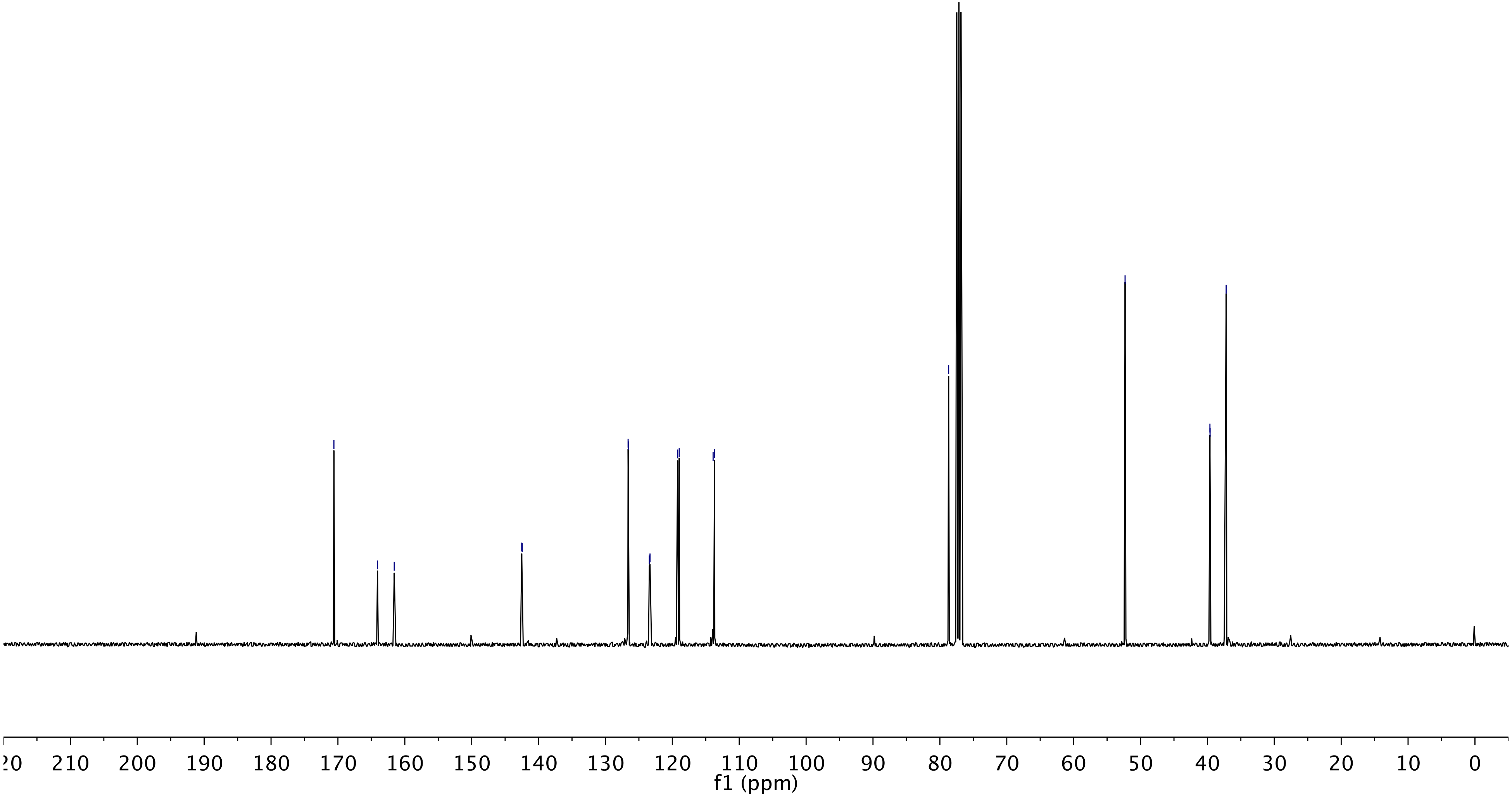

^{1}H NMR (400 MHz, $CDCl_3$)

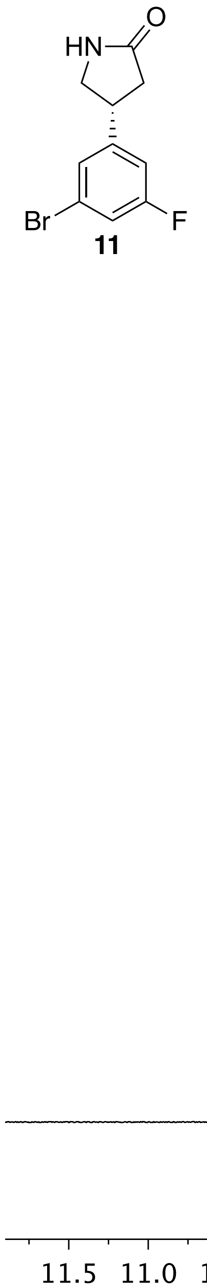

11

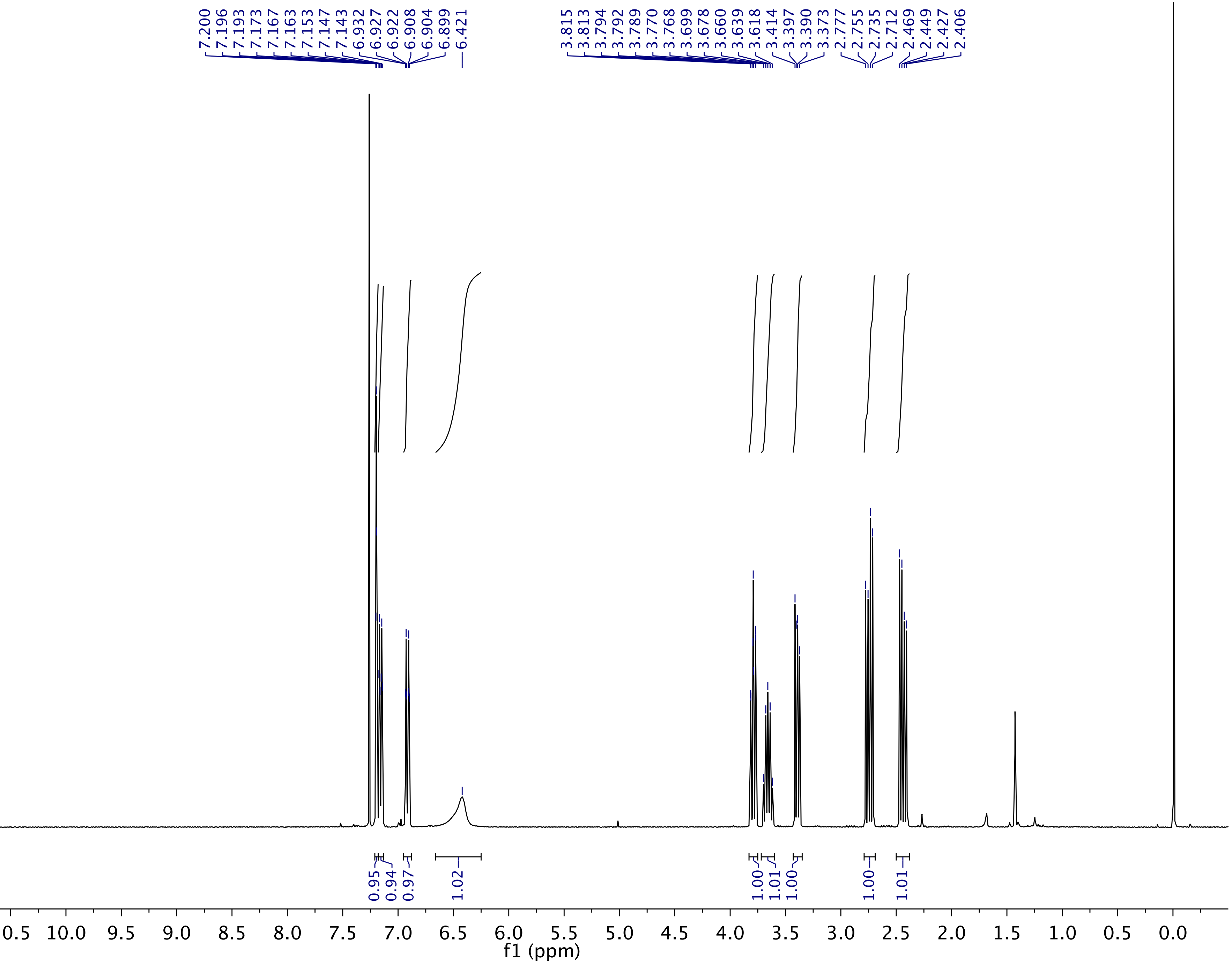

$^{13}C\{^{1}H\}$ NMR (101 MHz, $CDCl_3$)

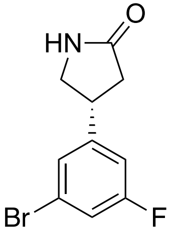

11

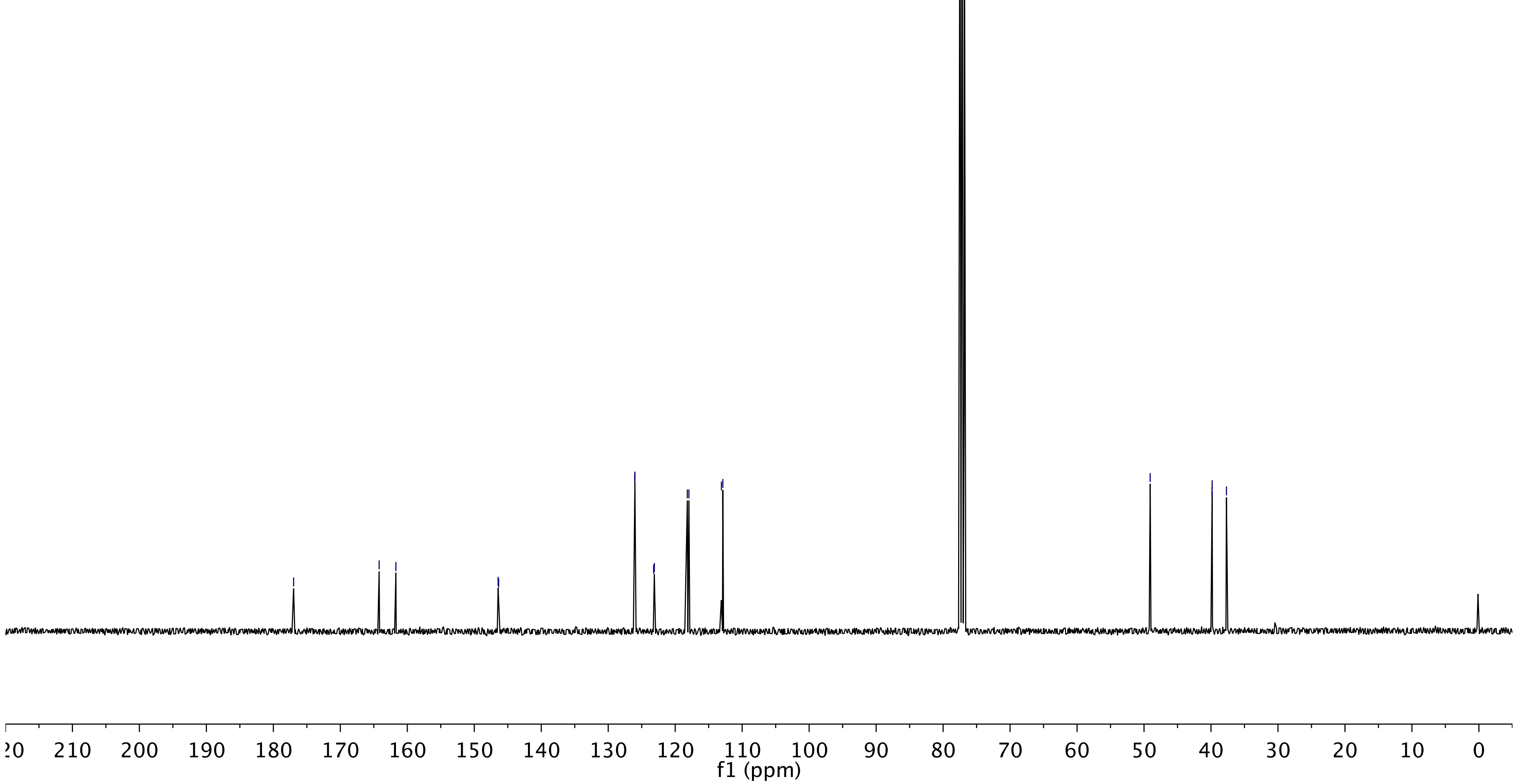

^{1}H NMR (400 MHz, $CDCl_3$)

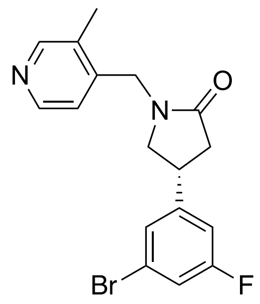

13

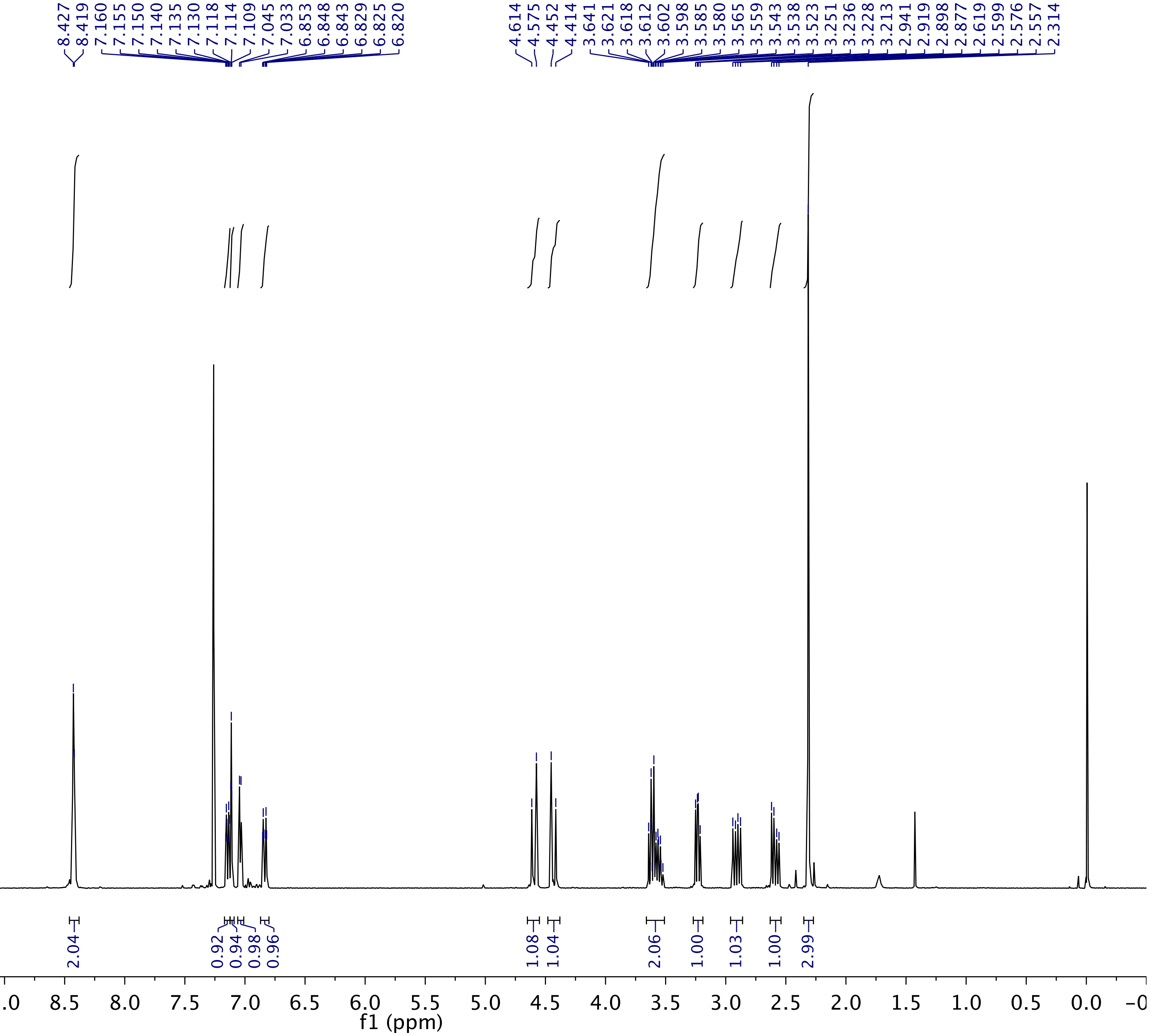

$^{13}C\{^1H\}$ NMR (101 MHz, $CDCl_3$)

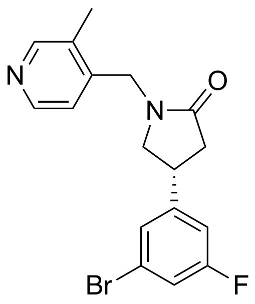

13

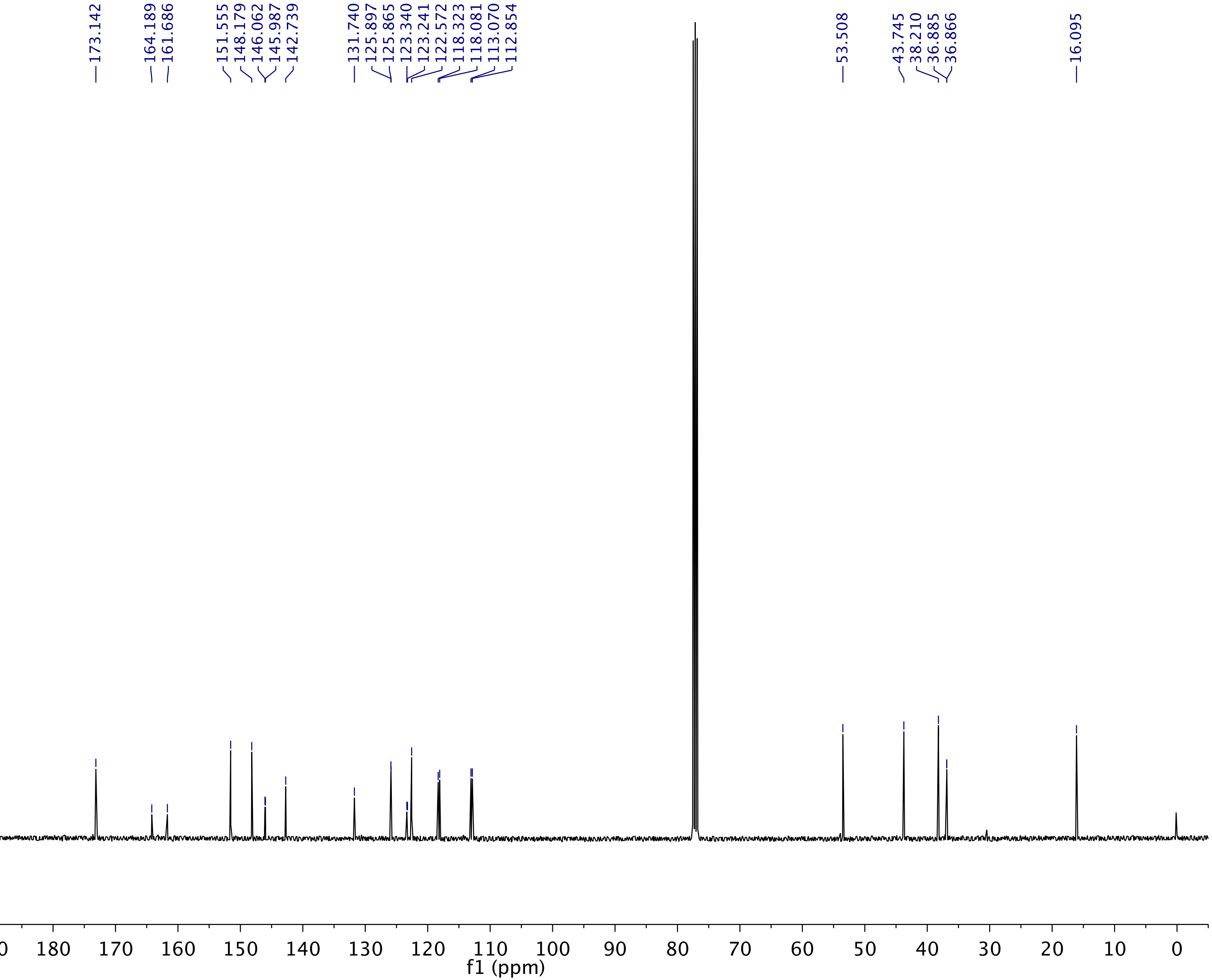

^{1}H NMR (400 MHz, $CDCl_3$)

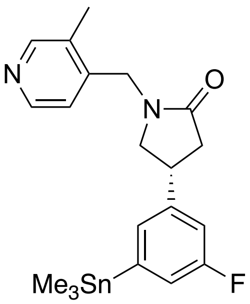

14

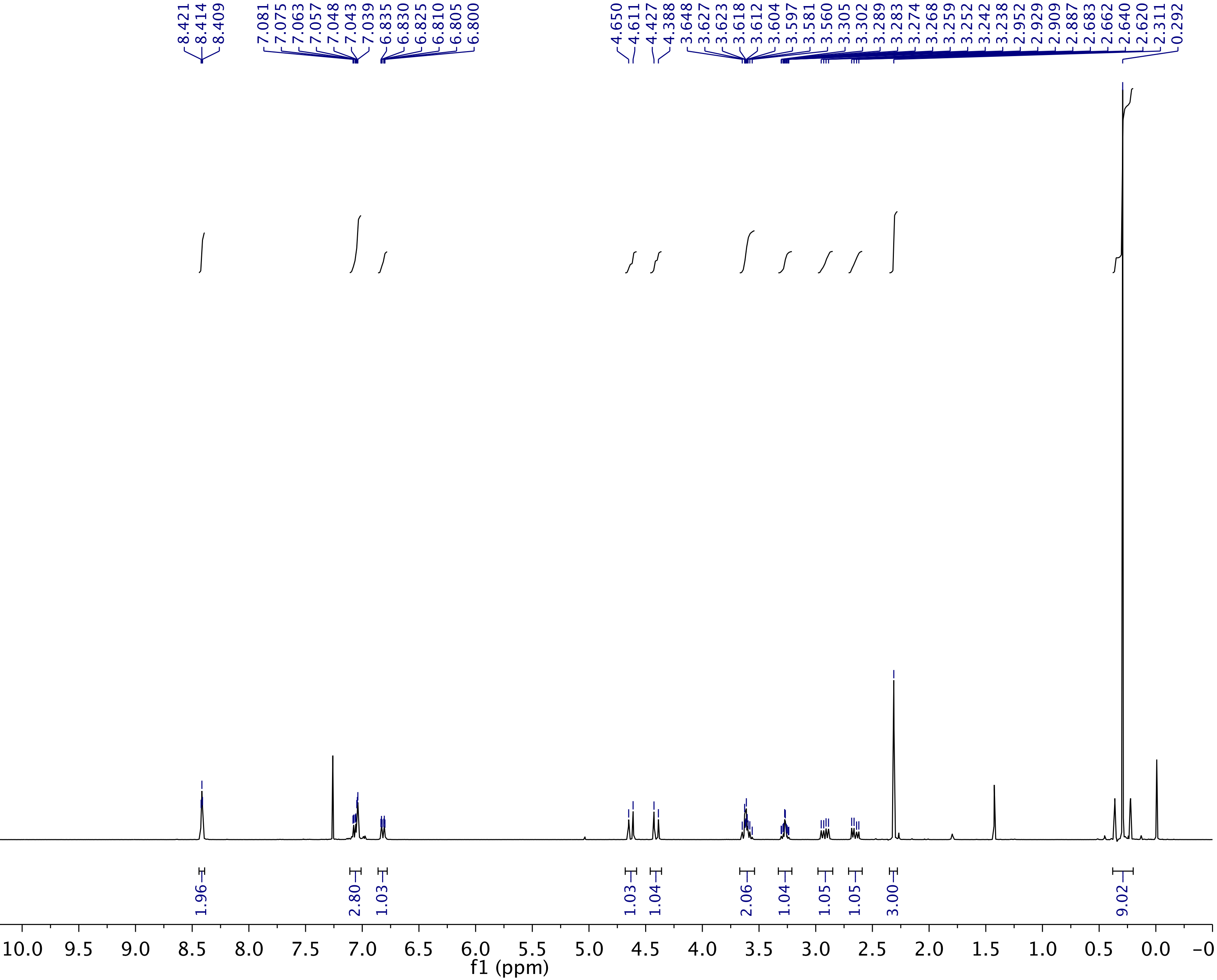

$^{13}C\{^{1}H\}$ NMR (101 MHz, $CDCl_3$)

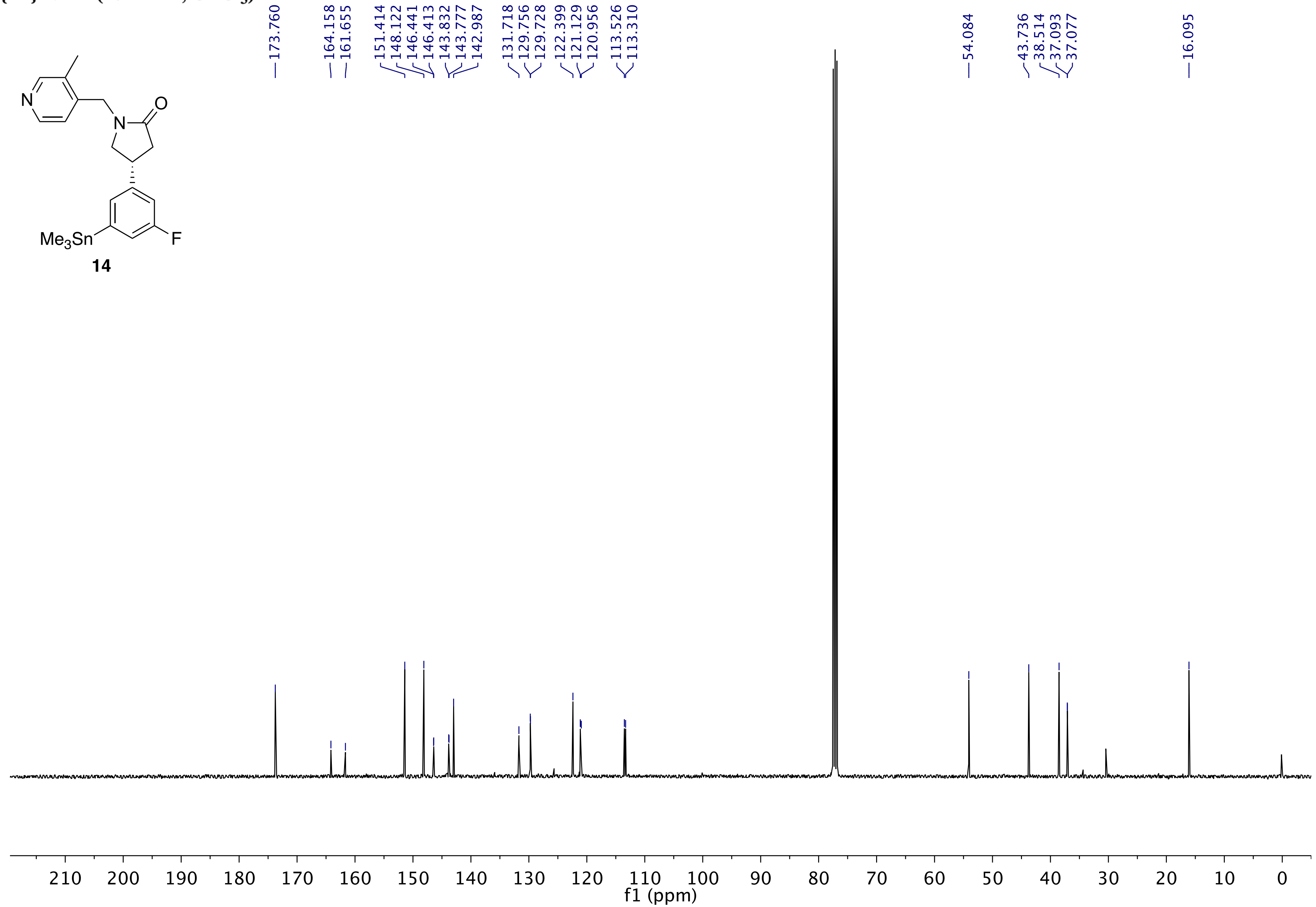

Supplement: Supplementary file 1 — jo2c01895_si_001.pdf [file jo2c01895_si_001.pdf]
